# Combined morphological and molecular data unveils relationships of *Pseudobranchiomma* (Sabellidae, Annelida) and reveals higher diversity of this intriguing group of fan worms in Australia, including potentially introduced species

**DOI:** 10.3897/zookeys.622.9420

**Published:** 2016-10-06

**Authors:** María Capa, Anna Murray

**Affiliations:** 1 NTNU University Museum, Norwegian University of Science and Technology, NO-7491 Trondheim, Norway; 2 Australian Museum Research Institute, 1 William St, Sydney, 2010, NSW, Australia

**Keywords:** new species, sabellids, feather duster worms, dichotomous key, taxonomy, invasive species, translocations

## Abstract

*Pseudobranchiomma* (Sabellidae, Annelida) is a small and heterogeneous group of fan worms found in shallow marine environments and is generally associated with hard substrates. The delineation and composition of this genus is problematic since it has been defined only by plesiomorphic characters that are widely distributed among other sabellids. In this study we have combined morphological and molecular (mitochondrial and nuclear DNA sequences) data to evaluate species diversity in Australia and assess the phylogenetic relationships of these and other related sabellids. Unlike morphological data alone, molecular data and combined datasets suggest monophyly of *Pseudobranchiomma*. In this study, a new species of *Pseudobranchiomma* is described and three others are considered as potential unintentional introductions to Australian waters, one of them reported for the first time for the continent. *Pseudobranchiomma pallida* **sp. n.** bears 4–6 serrations along the radiolar flanges, lacks radiolar eyes and has uncini with three transverse rows of teeth over the main fang. In the new species the colour pattern as well is characteristic and species specific.

## Introduction

*Pseudobranchiomma* Jones, 1962 (Sabellidae, Annelida) is a heterogeneous worldwide-distributed genus of fan worms inhabiting shallow marine habitats. Their tubes are made of muddy sediment embedded into a mucous matrix usually attached to hard substrates. Some species of *Pseudobranchiomma* are considered fouling organisms, settling on artificial surfaces and in some cases are common in harbour environments (Russell and Hewitt 2000, Swami and Udhayakumar 2010, Tovar-Hernández and Dean 2014). At least six species have been described as reproducing asexually, by fission of posterior segments, also known as scissiparity (Nogueira and Knight-Jones 2002, Knight-Jones and Giangrande 2003, Tovar-Hernández and Dean 2014). This reproductive strategy is not infrequent among sabellids (e.g. Knight-Jones and Bowden 1984; Fitzhugh 2003; Nogueira and Knight-Jones 2002; Nogueira et al. 2004, Tovar-Hernández and Knight-Jones 2006, Tovar-Hernández et al. 2009a, 2011, Kolbalsova et al. 2013) and allows them to colonise or form aggregations of clones in a short period of time under favourable conditions.

Due to these attributes, some members of *Pseudobranchiomma* are susceptible to being translocated by attachment to ship hulls, and may settle in new locations, if environmental factors permit. Unintentional translocations have been assessed and are well documented in members of the related genus *Branchiomma* (e.g. Knight-Jones et al. 1991, Licciano et al. 2002; Zenetos et al. 2005, 2010; Çinar et al. 2006; Tovar-Hernández and Knight-Jones 2006; El Haddad et al. 2008, Çinar 2009; Román et al. 2009, Tovar-Hernández et al. 2009a,b, 2011; Tovar-Hernández and Dean 2010, Arias et al. 2012; Giangrande et al. 2012, Capa et al. 2013). There is some indication that potential introduction of some *Pseudobranchiomma* species has already occurred in northern Australia (Russell and Hewitt 2000). For this reason, correct identification and assessment of potential introduced species is of great importance.

There are 16 species currently circumscribed within the genus (Nogueira and Knight-Jones 2002, Knight-Jones and Giangrande 2003, Tovar-Hernández and Dean 2014). The most conspicuous morphological interspecific differences within *Pseudobranchiomma* are the absence or presence of serrated radiolar flanges (paired lappets along the radioles lateral margins that may be few in number or extend along the full radiolar length), absence or presence of paired compound radiolar eyes, shape of the ventral collar glandular shield (rectangular, M-shaped, trapezoidal or divided in two), absence or presence of pinnular appendages, number of thoracic segments (Knight-Jones and Giangrande 2003), together with a broad variety of colour-morphs (Nogueira and Knight-Jones 2002, Knight-Jones and Giangrande 2003, Tovar-Hernández and Dean 2014). The monophyly of the genus has not yet been assessed. Species have been grouped into three artificial groups based on the absence, presence and relative length of the serrated radiolar flanges (Knight-Jones and Giangrande 2003): Group A, with radiolar serrations evenly distributed along the entire length of the radioles, or at least for most of their length; Group B, with serrations restricted to the distal part of the radioles; and Group C with smooth radiolar flanges.

Prior to the present study, *Pseudobranchiomma* has been reported from Australia as *Pseudobranchiomma orientalis* (McIntosh 1885), and *Pseudobranchiomma* cf. *Pseudobranchiomma emersoni* Jones 1962, in Northern Territory ports by Russell and Hewitt (2000). Capa and Murray (2015) also reported two species from the Great Barrier Reef, Queensland.

*Pseudobranchiomma* belongs to a group of sabellids possessing segmental eyespots between the noto- and neuropodia, spine-like chaetae arranged in oblique rows in the inferior thoracic fascicles, and well-developed conical abdominal neuropodia with chaetae arranged in C-shaped fascicles, together with *Bispira* Krøyer, 1856, *Branchiomma* Kölliker, 1858, *Sabella* Linnaeus, 1767, *Sabellastarte* Savigny, 1818 and *Stylomma* Knight-Jones, 1997 (e.g. Fitzhugh 1989, Fitzhugh and Rouse 1999; Capa 2008). *Branchiomma* and *Pseudobranchiomma* have long been considered to be closely related taxa due to the presence of stylodes (Jones 1962; Fitzhugh 1989) but the radiolar structures present in members of both genera were later considered not to be homologous (Knight-Jones 1994, Nogueira and Knight-Jones 2002, Knight-Jones and Giangrande 2003, Nogueira et al. 2006). Recently, other evidence of this close relationship has emerged, including the absence of companion chaetae, presence of four rows of vacuolated cells supporting the radioles, and a multicellular supporting axis of the radiolar appendages of the dorsal lips (Capa 2008, Capa et al. 2011).

The aims of this study are (1) to assess monophyly of *Pseudobranchiomma* and relationships with other members of the clade - *Bispira*, *Branchiomma*, *Sabella*, *Sabellastarte* and *Stylomma* - integrating morphological data and available mitochondrial and nuclear sequences; (2) to test whether the artificial groups proposed by Knight-Jones and Giangrande (2003) have some evolutionary meaning; (3) to assess species diversity in Australian waters and describe new species; (4) to provide a dichotomous key to enable identification of *Pseudobranchiomma* species.

## Methods

### Specimens and features examined

Fourteen *Pseudobranchiomma* terminals, including at least two species of each of the groups proposed by Knight-Jones and Giangrande (2003), were included in a morphological matrix, in order to assess their evolutionary relationships and test if these groups have any evolutionary meaning (i.e. natural groups). Members of other related genera including *Branchiomma*, *Sabella*, *Sabellastarte* and *Stylomma* were also incorporated in the analyses to test monophyly of *Pseudobranchiomma*, using *Pseudopotamilla* Bush, 1905 as the outgroup. The matrix (Table 1) was constructed in Mesquite (Maddison and Maddison 2015) and was scored after direct examination and using original descriptions and illustrations. Characters, states and scoring methods have been based on previous studies (e.g. Fitzhugh 1989, Fitzhugh and Rouse 1999, Capa 2008, Capa et al. 2010) or developed for the present study (see Table 2). The codification scheme included the presence or absence of traits and unordered multistate characters. Taxa lacking a feature were scored as inapplicable and indicated as a gap ‘-’ and unknown as a question mark ‘?’.

**Table 1.** Matrix of morphological character states (‘–’, inapplicable; ‘?’, uncertain/unknown, V, variable).

Species	1				5					10					15					20					25					30			33
*Pseudopotamilla* cf. *Pseudopotamilla reniformis*	0	0	0	-	0	0	1	1	1	1	0	1	0	1	0	1	0	0	2	0	0	1	0	1	0	0	1	1	1	1	0	1	?
*Bispira manicata*	0	1	1	0	1	0	1	1	3	0	-	0	1	0	1	0	1	2	2	1	1	1	0	1	1	1	0	0	1	0	0	0	1
*Bispira porifera*	0	1	0	-	0	0	1	0	-	1	0	0	1	1	1	0	1	0	2	1	1	1	0	1	1	1	0	0	1	0	0	0	?
*Bispira serrata*	0	1	1	1	0	0	1	1	3	1	0	0	1	1	1	0	1	0	2	1	1	0	0	1	1	1	0	0	0	0	0	1	?
*Branchiomma* sp. 1	0	0	0	-	1	1	0	1	3	0	-	0	1	1	1	0	1	0	0	1	1	0	1	0	1	1	0	0	0	0	0	1	1
*Branchiomma bairdi*	0	0	0	-	1	1	0	1	3	0	-	0	1	1	1	0	1	0	0	1	1	0	1	0	1	1	0	0	0	0	0	1	1
*Sabella spallanzanii*	1	1	0	-	1	0	0	0	-	0	-	0	1	0	1	0	1	0	1	1	1	1	0	1	1	1	0	0	1	0	0	0	1
*Sabella pavonina*	1	1	0	-	1	0	0	0	-	0	-	0	1	0	1	0	1	0	2	1	1	1	0	1	1	1	0	0	1	0	0	0	1
*Sabellastarte australiensis*	1	1	0	-	0	0	1	0	-	1	0	0	1	0	0	1	1	0	0	1	1	1	0	0	1	1	0	0	1	0	1	0	1
*Sabellastarte* sp.	1	1	0	-	0	0	1	0	-	1	0	0	1	0	0	1	1	0	0	1	1	1	0	0	1	1	0	0	1	0	1	0	1
*Stylomma palmatum*	0	1	1	0	0	0	1	1	2	1	1	0	0	0	0	0	1	0	0	1	1	1	0	1	1	1	0	0	1	0	0	0	?
*Stylomma juani*	0	1	1	1	0	0	1	1	0	1	1	0	0	0	0	0	1	0	0	1	1	1	0	1	1	1	0	0	1	0	0	0	?
*Pseudobranchiomma emersoni*	0	0	1	1	?	0	0	0	-	0	-	0	1	0	1	0	1	1	2	1	1	0	1	0	1	1	0	0	0	0	0	1	1
*Pseudobranchiomma* cf. *Pseudobranchiomma emersoni* (Australia)	0	0	1	1	1	0	0	0	-	0	-	0	1	0	1	0	1	1	2	1	1	0	1	0	1	1	0	0	0	0	0	1	?
*Pseudobranchiomma minima*	0	0	0	-	1	0	0	0	-	0	-	0	1	0	1	0	1	1	1	1	1	0	1	0	1	1	0	0	0	0	0	?	1
*Pseudobranchiomma orientalis*	0	1	1	1	1	0	?	0	-	0	-	0	1	0	1	0	1	0	2	1	1	0	1	0	1	1	0	0	0	0	0	1	?
*Pseudobranchiomma* cf. *Pseudobranchiomma orientalis* (Australia)	0	1	1	1	1	0	1	0	-	0	-	0	1	0	1	0	1	0	2	1	1	0	1	0	1	1	0	0	0	0	0	1	?
*Pseudobranchiomma pallida* sp. n.	0	0	1	1	0	0	0	0	-	0	-	0	1	0	1	0	1	1	1	1	1	0	1	0	1	1	0	0	0	0	0	?	?
*Pseudobranchiomma paraemersoni*	0	0	1	1	1	0	0	0	-	0	-	0	1	0	1	0	1	1	2	1	1	0	1	0	1	1	0	0	0	0	0	?	?
*Pseudobranchiomma paulista*	0	0	1	1	1	0	0	0	-	0	-	0	1	?	1	0	1	V	2	1	1	0	1	0	1	1	0	0	0	0	0	?	?
*Pseudobranchiomma perkinsi*	0	0	0	-	0	0	0	1	3	0	-	0	1	0	1	0	1	2	2	1	1	0	1	0	1	1	0	0	0	0	0	1	1
*Pseudobranchiomma punctata*	0	1	0	-	1	0	1	0	-	1	0	0	1	0	1	1	1	1	0	1	1	0	1	0	1	1	0	0	0	0	0	?	1
*Pseudobranchiomma serratibranchis*	0	0	1	1	1	0	0	?	?	?	-	0	1	0	1	0	1	0	0	1	1	?	1	0	1	1	0	0	0	0	0	?	?
*Pseudobranchiomma schizogenica*	0	1	1	1	1	0	?	0	-	0	-	0	1	0	1	0	1	1	2	1	1	0	1	0	1	1	0	0	0	0	0	1	1
*Pseudobranchiomma* cf. *Pseudobranchiomma schizogenica* (Australia)	0	1	1	1	1	0	0	0	-	0	-	0	1	0	1	0	1	1	2	1	1	0	1	0	1	1	0	0	0	0	0	1	1
*Pseudobranchiomma tarantoensis*	0	0	0	-	1	0	1	0	-	0	-	0	1	0	1	0	1	0	1	1	1	1	1	0	1	1	0	0	0	0	0	1	0

**Table 2.** Morphological characters and states scored in the matrix (Table 1). These are based on previous cladistic analyses (e.g. Fitzhugh and Rouse 1999, Capa 2008, Capa et al. 2010), in addition to other features consider diagnostic for *Pseudobranchiomma* species.

1.	*Lobes*: (0) semicircular or involuted; (1) spiral.
2.	*Basal membrane*: (0) absent (or reduced, shorter than one thoracic segment); (1) present (longer than one thoracic segment).
3.	*Radiolar flanges*: (0) absent or reduced to ridges; (1) present.
4.	*Serrations of radiolar flanges*: (0) absent; (1) present.
5.	*Transverse pigment bands on radioles*: (0) absent; (1) present.
6.	*Stylodes*: (0) absent; (1) present.
7.	*Number of rows of vacuolated cells supporting radioles*: (0) four; (1) more than four.
8.	*Radiolar eyes*: (0) absent; (1) present.
9.	*Radiolar eyes arrangement*: (0) ocelli; (1) unpaired proximal compound eyes on radiole dorsal margin; (2) unpaired terminal compound eyes on inner peduncule; (3) paired compound eyes;
10.	*Dorsal basal flanges*: (0) absent; (1) present.
11.	*“Press-stud” structure present on dorsal basal flanges*: (0) absent; (1) present.
12.	*Ventral basal flanges*: (0) absent; (1) present.
13.	*Dorsal lip radiolar appendage vacuolated cells (skeleton)*: (0) absent; (1) present.
14.	*Dorsal pinnular appendages*: (0) absent; (1) present.
15.	*Position of ventral sacs*: (0) inside the radiolar crown; (1) outside the radiolar crown.
16.	*Dorsal margin of posterior peristomial ring collar*: (0) widely separated; (1) fused to the faecal groove.
17.	*Interramal eyespots*: (0) absent; (1) present.
18.	*Number of thoracic segments*: (0) 8; (1) generally less than 8; (2) generally more than 8.
19.	*Gap between thoracic tori and ventral shields*: (0) absent, all ventral shields in contact with tori; (1) gap between ventral shields and tori of anterior thoracic segments; (2) present, gap in all thoracic segments.
20.	*Thoracic chaetal fascicles (notopodia)*: (0) transverse rows; (1) longitudinal bundles.
21.	*Inferior thoracic notochaetae shape*: (0) paleate; (1) spine-like.
22.	*Rows of teeth on thoracic uncini*: (0) few (1–5); (1) numerous (>5).
23.	*Length of thoracic uncini handles*: (0) medium length (more than distance from breast to tip of main fang); (1) short (shorter than distance of breast to tip of main fang).
24.	Thoracic *companion chaetae*: (0) absent; (1) present.
25.	*Abdominal neurochaetal tori*: (0) transverse ridges; (1) conical lobes.
26.	*Abdominal neurochaetal fascicles*: (0) transverse row(s); (1) spiralled.
27.	*Superior row of abdominal neurochaetal fascicles*: (0) elongated narrowly hooded; (1) broadly hooded.
28.	*Inferior row of abdominal chaetae*: (0) spine-like; (1) broadly hooded.
29.	*Abdominal uncini number of rows*: (0) few (1–5); (1) numerous (>5).
30.	*Abdominal uncini breast*: (0) well developed, expanded; (1) narrow, swelling.
31.	*Length of abdominal uncini handles*: (0) short (shorter than distance of breast to tip of main fang); (1) medium (1–2 times distance of breast to tip of main fang).
32.	*Pygidial shape*: (0) rim; (1) bilobed.
33.	*Scissiparity*: (0) absent; (1) present.

More than 100 specimens deposited in Australian museum collections were examined and identified to species to assess the species diversity in Australian waters. These included specimens identified for the Darwin Ports Survey and mentioned in the report by Russell and Hewitt (2000) which had been deposited at the Museum and Art Gallery of the Northern Territory (NTM). Type material of herein newly described species was deposited in the Australian Museum
(AM). After study of all specimens with stereo microscopy, some parapodia (typically from mid-thoracic as well as abdominal regions) were removed and mounted with glycerine on slides for studying thoracic and abdominal chaetae. Line drawings were made to scale with a drawing tube attached to a Zeiss MI compound microscope. Final drawings were created using Adobe Illustrator© software. Descriptions in the text of relative dimensions of chaetal features are based on the terminology used by Nogueira et al. (2006). Some specimens were stained with methyl green to reveal thoracic glandular patterns. Other specimens were dehydrated in ethanol, critical-point dried, covered with 20 nm of gold and examined under a Leo 435VP scanning electron microscope at the Australian Museum, using ET secondary electron detector. A detailed morphological comparison was performed between species considered as Group A of Knight-Jones and Giangrande (2003), in order to find out clear differences between species (Table 3).

**Table 3.** Comparison of species from Group A (according to Knight-Jones and Giangrande 2003), possessing serrations along the radiolar flanges. Information retrieved from original descriptions except where indicated. ? = information not available.

TAXON	Distribution	Radiolar eyes	Radioles (pairs)	Radiolar serrations (pairs)	Radiolar lobes	Dorsal lips/crown length	Crown pigment	Body pigment alive (preserved)	Interramal eyespots	Thoracic tori and ventral shields	Thoracic segments	Teeth thoracic uncini
*Pseudobranchiomma emersoni* Jones, 1962	Jamaica, Florida (USA), Cape Verde Islands?	no	14^†^	10	semicircular?	1/4	irregular	brownish with spots (same)	large^†^	gap^†^	4–6 (8^‡^)	5 (5–6)^†^
*Pseudobranchiomma* cf. *Pseudobranchiomma emersoni*	Queensland, Australia	no	17	10	semicircular	1/6	irregular	pale with large anterior spots	small	gap	7	3
*Pseudobranchiomma grandis* (Baird, 1865)	New Zealand	yes^‡, §, ¶^	>10 (≤30)^¶^	? (>18)^¶^	semicircular	?	9 (4–9)^¶^ irregular bands	pale with brown/purple spots^¶^ (dark brown)	small^¶^	Almost in contact^¶^	7 (8)^¶^	?
*Pseudobranchiomma serratibranchis* (Grube, 1878)	Philippines	yes^‡, §^	17	30	semicircular-	?	7–8 bands	?	?	?	8	?
*Pseudobranchiomma orientalis* (McIntosh, 1885)	Hong Kong	no	26	13–16^†^	involuted	1/4^†^	23 bands	internal purple-brown spots on ventral lappets (colourless^#^)	present only in abdomen	gap^#^	8^†,#^	6–7 (4–5)^†^
*Pseudobranchiomma* cf. *Pseudobranchiomma orientalis*	Queensland, and Northern Territory, Australia	no	10–30	10–20	involuted~1 whorl	1/6–1/3	10–20 bands	(pale with few anterior spots)	tiny	gap	7–8	5–7
*Pseudobranchiomma paraemersoni* Nogueira et al., 2006	São Paulo, Brazil	no	6	3–4	semicircular	1/4	3–4 bands	bright yellow with few spots (white)	large	gap	4–5	4–5
*Pseudobranchiomma paulista* Nogueira et al., 2006	São Paulo, Brazil	no	22–25	13–19	semicircular	1/6	10–19 bands	pale yellow with purple spots (white with spots)	small	gap	6–10	4–5
*Pseudobranchiomma pallida* sp. n.	Queensland, Australia	no	9	4–5	semicircular	1/6	No banding	colourless	large	gap	7	3
*Pseudobranchiomma schizogenica* Tovar-Hernández and Dean, 2014	Gulf of California, Mexico	no	6–9	6–11	semicircular	1/4	4 or more purple bands with orange and translucent bands between	bright yellow with few purple spots (pale yellow)	large	?	5	4
*Pseudobranchiomma* cf. *Pseudobranchiomma schizogenica*	North Australia and Hawaii	no	9	4–6	semicircular	1/3	4–6 bands and orange band in base	pale with spots	large	gap	4–7	3

According to: † Tovar-Hernández and Dean 2014; ‡ Knight-Jones and Giangrande 2003; § Nogueira et al. 2006; ¶ Geoff Read, pers. comm.; # Knight-Jones unpublished drawings and description of the type.

### Molecular data

Genomic DNA was extracted from sample tissue using standard protocols for the DNeasy Animal tissues protocol (manufactured by QIAGEN Pty Ltd). Sections of two mitochondrial genes cytochrome b (cob) and cytochrome oxidase I (cox1), and one nuclear gene ribosomal internal transcribed spacer 1 (ITS1), were then amplified using the primers Cytb 424F (RT-1) and cobr825 (Burnette et al. 2005) for cob, HCO2198 and LCO1490 (Folmer et al. 1994) for cox1and ITSF (Chen et al. 2002) and ITSR1 (Capa et al. 2011) for ITS1 (Table 4). Standard PCR conditions (carried out in 25ul volumes containing 2.5uL of QIAGEN 10x PCR buffer, 1.5mM MgCl2, 0.05mM of each dNTP, 10 pmol of each primer, 1 unit of QIAGEN Taq DNA polymerase, and 1–100ng of whole genomic DNA). A negative control (containing no DNA template) is included for each batch of amplifications to exclude the possibility that any results achieved are due to contaminant DNA. Amplifications were performed on a MastercyclerS Gradient (Eppendorf Inc). The PCR thermal cycling profile was 94°C for 2 min, followed by 35 cycles of 94°C for 20 sec, 52°C for 40 sec, 72°C for 1 min and a 5 min final extension at 72°C. Successful amplifications were then purified using the ExoSAP-IT PCR purification (USB Corporation) system, then bi-directionally sequenced, using the original PCR primers, at an external sequencing facility. Chromatographs were annotated with the program SEQUENCHER v. 5.1 (Gene Codes Corporation). ITS1 sequence chromatograms showed no evidence of double peaks, suggesting the presence of multiple copies.

**Table 4.** Taxa and GenBank accession numbers for the genes sequenced for the present study. NSW, New South Wales; NT, Northern Territory, QLD, Queensland; SA, South Australia; WA, Western Australia.

Taxon	Voucher	cox1	cob	ITS1	Locality
*Pseudopotamilla* cf. *Pseudopotamilla reniformis*	AM W.36444	KX894903	KX894900	KX894909	Darwin, NT, Australia
*Branchiomma* sp.	AM W.35580	–	KF429111	KX894915	Oahu, Hawaii, USA
*Branchiomma bairdi*	AM W.31822	KP254646	KF429105	KF459971	Fort Pierce, Florida, USA
*Bispira serrata*	AM W.36979	KX894907	–	KX894916	Lizard Island, QLD, Australia
*Bispira manicata*	AM W.36964	KX894904	KX894902	KX894910	Aquarium at Oceanword, NSW, Australia
*Sabella spallanzanii*	AM W.30505	KX894905	–	–	SA, Australia
*Stylomma palmatum*	AM W.36959	KX894908	KX894901	KX894914	Ningaloo Reef, WA, Australia
*Sabellastarte australiensis*	AM W.35608	–	KF429134	KF460007	Cape Banks, NSW, Australia
*Sabellastarte* sp.	AM W.36977	KX894906	–	KX894913	Port Phillip Bay, VIC, Australia
*Pseudobranchiomma pallida* sp. n.	AM W.36366	–	–	KX894911	Heron Island, QLD, Australia
*Pseudobranchiomma* cf. *Pseudobranchiomma schizogenica* (Australia)	AM W.36364	–	–	KX894912	Heron Island, QLD, Australia
*Pseudobranchiomma* cf. *Pseudobranchiomma schizogenica* (Hawaii)	AM W.35576	–	KF429108	KF459975	Oahu, Hawaii, USA

### DNA sequence alignments

Nucleotide sequences of *cob*, *cox1* and ITS1 were aligned with MAFFT v. 6.0 (Katoh 2013) using default parameters, in all cases auto-selected strategy was L-INS-i. Additionally, ITS1 sequences was also aligned using the Q-INS-i algorithm that takes into account secondary structure. Poorly aligned positions from divergent regions of ITS1 were removed using GBLOCKS v. 0.91b with relaxed parameters (minimum number of sequences for a conserved position: 7, minimum number of sequences for a flanking position: 7, maximum number of contiguous non-conserved positions: 8, minimum length of a block: 5) to assess the impact of ambiguously aligned regions on the phylogenetic signal (Talavera and Castresana 2007). Matrices were concatenated in Mesquite (Maddison and Maddison 2015).

### Phylogenetic analyses


 Maximum parsimony (MP) heuristic searches used 10,000 replicates of random taxon addition and tree bisection-reconnection (TBR) branch swapping algorithm, saving 100 trees per replicate using TNT 1.1 (Goloboff et al. 2008a). All characters were given equal weight and multistate characters considered non-additive. Nodal support was estimated by 1,000 jackknife replicates using TBR, in TNT 1.1 (Goloboff et al. 2008a). New technology searches, such as ratchet, drift and tree fusing were implemented, isolated and in combination using TNT 1.1 (Goloboff et al. 2008a) performing 1,000 repetitions and hitting the most parsimonious trees 100 times. Tree metrics are abbreviated as follows: tree length (TL), consistency index excluding parsimony non-informative characters in the data matrix (CI), and retention index (RI). Support values are given on the trees. In order to reach a topology that better explains those characters with a better fit to the cladistic hypothesis, at the expense of the more homoplasious ones we have implemented implied weighting (Goloboff 1993, 1995, Goloboff et al. 2008a, b). With this method, a higher weight is given to those characters with less homoplasy, producing a much more resolved estimated consensus tree (Goloboff et al. 2008b). Results using a range of concavities (values for k) have been compared.


 Maximum likelihood (ML) analyses were conducted using RAxML (Stamatakis et al. 2008). All analyses were performed for the morphological data (Tables 1 and 5) and each marker independently and in combination (molecular data and molecular + morphological data), and with and without the poorly aligned positions from divergent regions of ITS1, using GBLOCKS.

**Table 5.** Morphological matrix for the specimens with DNA sequence data.

**Species**	**1**				**5**					**10**					**15**					**20**					**25**					**30**			**33**
Pseudopotamilla cf reniformis	0	0	0	-	0	0	1	1	1	1	0	1	0	1	0	1	0	0	2	0	0	1	0	1	0	0	1	1	1	1	0	1	?
*Bispira manicata*	0	1	1	0	1	0	1	1	3	0	-	0	1	0	1	0	1	2	2	1	1	1	0	1	1	1	0	0	1	0	0	0	1
*Bispira serrata*	0	1	1	1	0	0	1	1	3	1	0	0	1	1	1	0	1	0	2	1	1	0	0	1	1	1	0	0	0	0	0	1	?
*Branchiomma* sp.	0	0	0	-	1	1	0	1	3	0	-	0	1	1	1	0	1	0	0	1	1	0	1	0	1	1	0	0	0	0	0	1	1
*Branchiomma bairdi*	0	0	0	-	1	1	0	1	3	0	-	0	1	1	1	0	1	0	0	1	1	0	1	0	1	1	0	0	0	0	0	1	1
*Sabella spallanzanii*	1	1	0	-	1	0	0	0	-	0	-	0	1	0	1	0	1	0	1	1	1	1	0	1	1	1	0	0	1	0	0	0	1
*Sabellastarte* sp.	1	1	0	-	0	0	1	0	-	1	0	0	1	0	0	1	1	0	0	1	1	1	0	0	1	1	0	0	1	0	1	0	1
*Stylomma palmatum*	0	1	1	0	0	0	1	1	2	1	1	0	0	0	0	0	1	0	0	1	1	1	0	1	1	1	0	0	1	0	0	0	?
*Pseudobranchiomma pallida* sp. n.	0	0	1	1	0	0	0	0	-	0	-	0	1	0	1	0	1	1	1	1	1	0	1	0	1	1	0	0	0	0	0	?	?
*Pseudobranchiomma* cf *Pseudobranchiomma schizogenica* (Australia)	0	1	1	1	1	0	0	0	-	0	-	0	1	0	1	0	1	1	2	1	1	0	1	0	1	1	0	0	0	0	0	1	1
*Pseudobranchiomma* cf *Pseudobranchiomma schizogenica* (Hawaii)	0	1	1	1	1	0	0	0	-	0	-	0	1	0	1	0	1	1	2	1	1	0	1	0	1	1	0	0	0	0	0	1	1

## Results

### *Pseudobranchiomma* monophyly and relationships

**Figure 1.**
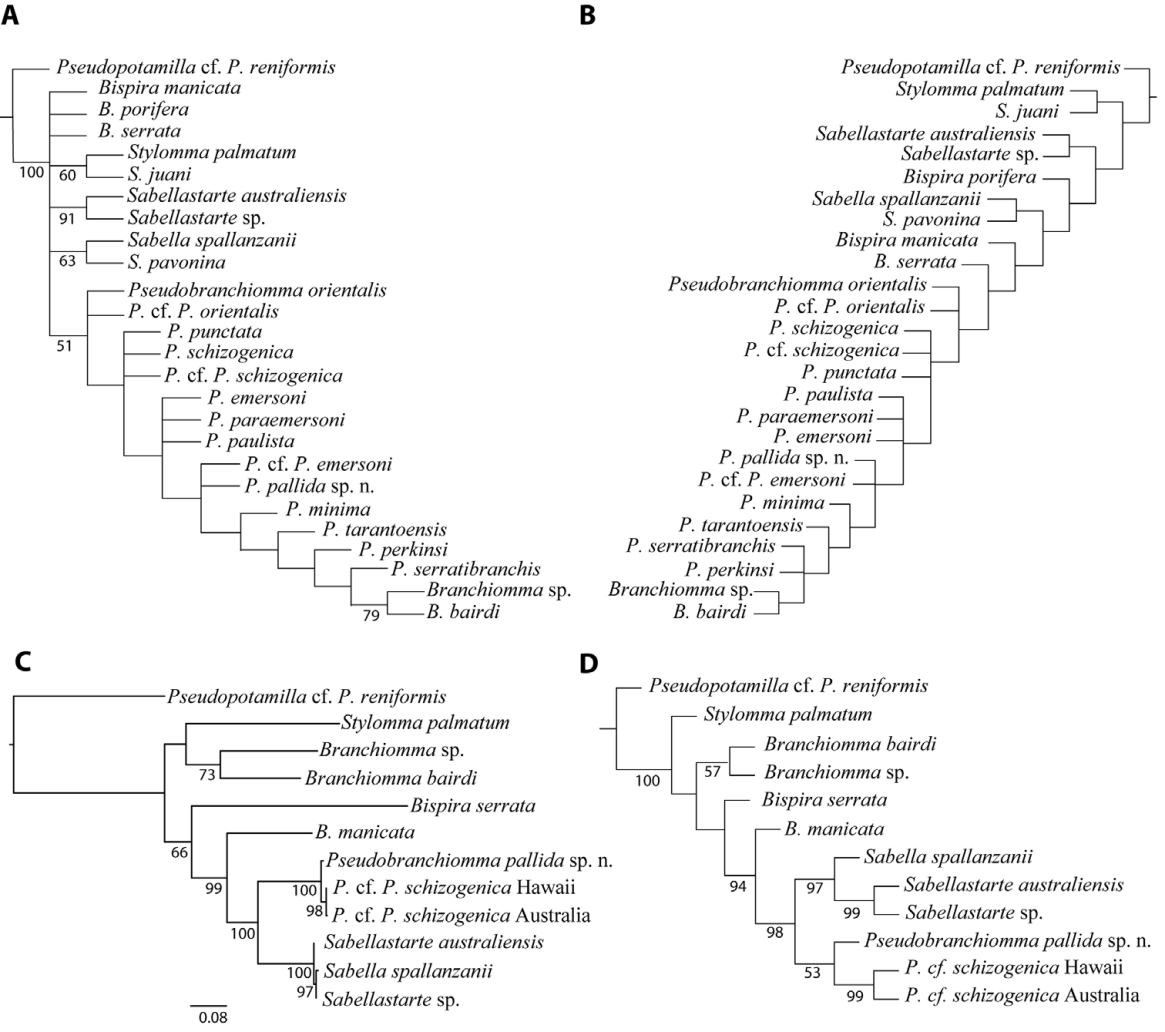
Phylogenetic hypothesis of *Pseudobranchiomma* and related taxa. **A** Strict consensus of six most-parsimonious trees after analyses of morphological data (33 characters) and 26 members of Sabellidae rooted with *Pseudopotamilla*. Jack-knife support values are given (>50) **B** Strict consensus of three most parsimonious tree after implementation of implied weighting (constant of concavity k = 7) **C** Tree after maximum likelihood analyses of mitochondrial and nuclear dataset. Bootstrap values on nodes if >50; scale: average of nucleotide substitutions per site **D** Single most-parsimonious tree after analyses of the combined morphological and molecular datasets (12 taxa and 2239 characters); Jack-knife support values are given (>50).

Maximum parsimony analyses of the complete morphological matrix (Table 1), including 26 terminals (14 terminals of *Pseudobranchiomma* and 12 species of other related sabellids) and 33 characters, all of which were parsimony-informative, yielded six most parsimonious trees (TL 69, CI 0.53, RI 0.75, Fig. 1A). *Branchiomma* was recovered nested within *Pseudobranchiomma* in all of these trees. Several polytomies within *Pseudobranchiomma* and between other related sabellids in the consensus tree, together with the low consistency index value, reflects the large amount of homoplasy in the dataset for resolving bifurcating branching pattern (Fig. 1A). Implied weighting with concavity of k = 3–6 recovered six most-parsimonious trees. Similar analyses with k values of 7–8 yielded three most parsimonious trees (Fig. 1B). In any of these topologies, monophyly of *Pseudobranchiomma* was assessed because *Branchiomma* was nested within the *Pseudobranchiomma* terminals. The few clades found within *Pseudobranchiomma* are not well supported. However, they do not concur with the groups proposed by Knight-Jones and Giangrande (2003), based on the presence and serration of the radiolar flanges, indicating these could be artificial.

The low success obtaining sequences out of tissue from members of *Pseudobranchiomma* (only three terminals belonging to two morphospecies), restricted the outcomes of the present study. Analyses of the combined molecular dataset yielded a phylogenetic hypothesis where *Pseudobranchiomma* was monophyletic and sister to *Sabella* + *Sabellastarte* (Fig. 1C). The same topology was recovered after the elimination of poorly aligned positions from divergent regions of ITS1 (not shown). Combination of morphological and molecular data (2239 characters, of which 783 were parsimony-informative, and 12 taxa) yielded a single most parsimonious tree (TL 2459, CI 0.76, RI 0.54; Fig. 1D) having the *Pseudobranchiomma* terminals in one clade, sister to *Sabella* + *Sabellastarte*. The two representatives of *Bispira* were not recovered as monophyletic (Fig. 1D) similar to the results obtained after analyses of morphological and molecular data alone (Fig. 1A–C). The combined dataset recovers *Branchiommma* and *Stylomma* branching off at the base of the ingroup (Fig. 1D).

### Taxonomy

#### Pseudobranchiomma

Taxon classificationAnimaliaSabellidaSabellidae

Jones, 1962

Pseudobranchiomma Jones, 1962: 198–201, figs 115–124; Fitzhugh 1989: 73; Nogueira and Knight-Jones 2002: 1661–1670; Knight-Jones and Giangrande 2003: 95–103; Nogueira et al. 2006: 588.

##### Type species.

*Pseudobranchiomma emersoni* Jones, 1962.

##### Diagnosis.

Radioles with or without radiolar flanges, serrated or smooth. Some species with paired compound eyes along radioles. Four rows of vacuolated cells supporting the radioles; a multicellular supporting axis of the radiolar appendages of the dorsal lips. Ventral sacs located outside the radiolar crown. Dorsal margins of collar separated from the faecal groove by a wide gap and without “pockets”. Segmental eyespots between the noto- and neuropodia. Spine-like chaetae arranged in oblique rows in the inferior thoracic fascicles. Thoracic companion chaetae absent. Well-developed conical abdominal neuropodia with chaetae arranged in C-shaped fascicles.

##### Remarks.

There is no apparent morphological synapomorphy supporting *Pseudobranchiomma*. The monophyly of the genus *Pseudobranchiomma* has not been tested prior to this study. The group has been defined by a combination of homoplastic characters: presence of radiolar flanges (shared with *Stylomma* and some *Bispira* species but absent in *Pseudobranchiomma longa* (Kinberg, 1867)); ventral sacs located outside the radiolar crown (shared with *Bispira*, *Branchiomma* and *Sabella*); dorsal margins of the collar separated from the faecal groove by a wide gap and without “pockets” (shared by *Bispira*, *Stylomma* and some species of *Branchiomma*); and absence of thoracic companion chaetae (shared with *Branchiomma* and *Sabellastarte*) (e.g. Knight-Jones 1994, Nogueira and Knight-Jones 2002, Nogueira et al. 2006). It has been suggested that the colour pattern of the crown, consisting of transverse yellow and purple bands, could be another diagnostic feature for the genus (Nogueira et al. 2006), but this feature is also very common in *Sabellastarte* species (e.g. Capa et al. 2010). The monophyly of *Pseudobranchiomma* is herein also questioned. Morphological data analysis recovers the group as paraphyletic, and the scarcity of molecular data gathered for the present study does not allow us to properly assess monophyly.

#### Pseudobranchiomma cf. emersoni

Taxon classificationAnimaliaSabellidaSabellidae

Jones, 1962

Figures 2 , 3A–F

? Pseudobranchiomma emersoni Jones, 1962:198–201, figs 115–124; Knight-Jones 1994: fig. 4j; Knight-Jones and Giangrande 2003: fig. 1 c–f; Tovar-Hernández and Dean 2014: 936, table 1.

##### Material examined.

**Australia: Queensland**: AM W.36365, (1 spec.), Heron Island, First Point, North Heron Reef, 23°25'48"S, 151°55'48"E, in coral rubble, 13 m, 12 Nov 2009.

##### Diagnosis.

Ten pairs of short flat radiolar serrations evenly distributed along entire length of radioles. RRadiolar eyes absent. Small gap between anterior thoracic ventral shields and neuropodial tori. Thoracic and abdominal uncini with five transverse rows of teeth surmounting main fang. Radiolar crown with wide purple band at base, irregular transverse purple bands on radioles and flanges and yellow band on distal end of radioles. Body pale with distinct interramal eyespots and purple pigment spots on thorax and dorsally on abdomen.

##### Description of Australian specimen.

**Figure 2.**
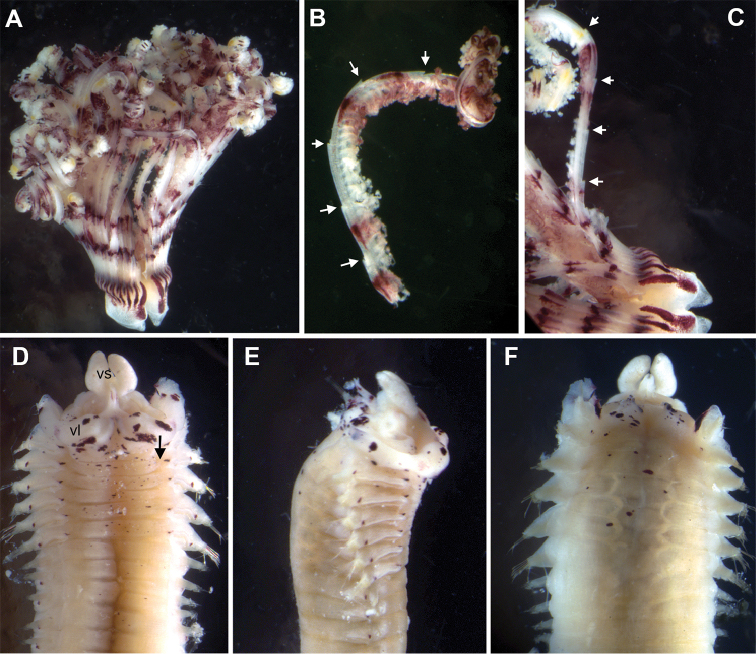
*Pseudobranchiomma* cf. *Pseudobranchiomma emersoni* AM W.36365; photographs of preserved specimen; **A** Radiolar crown, dorsal view **B** Detached radiole **C** Detail of base of crown and single radiole **D** Anterior segments, ventral view (crown detached) **E** Same, lateral view **F** Same, dorsal view. vl, ventral lappet; vs, ventral sacs; white arrows, serrations of radiolar lateral flanges; black arrow, gap between ventral shields and thoracic tori.

**Figure 3.**
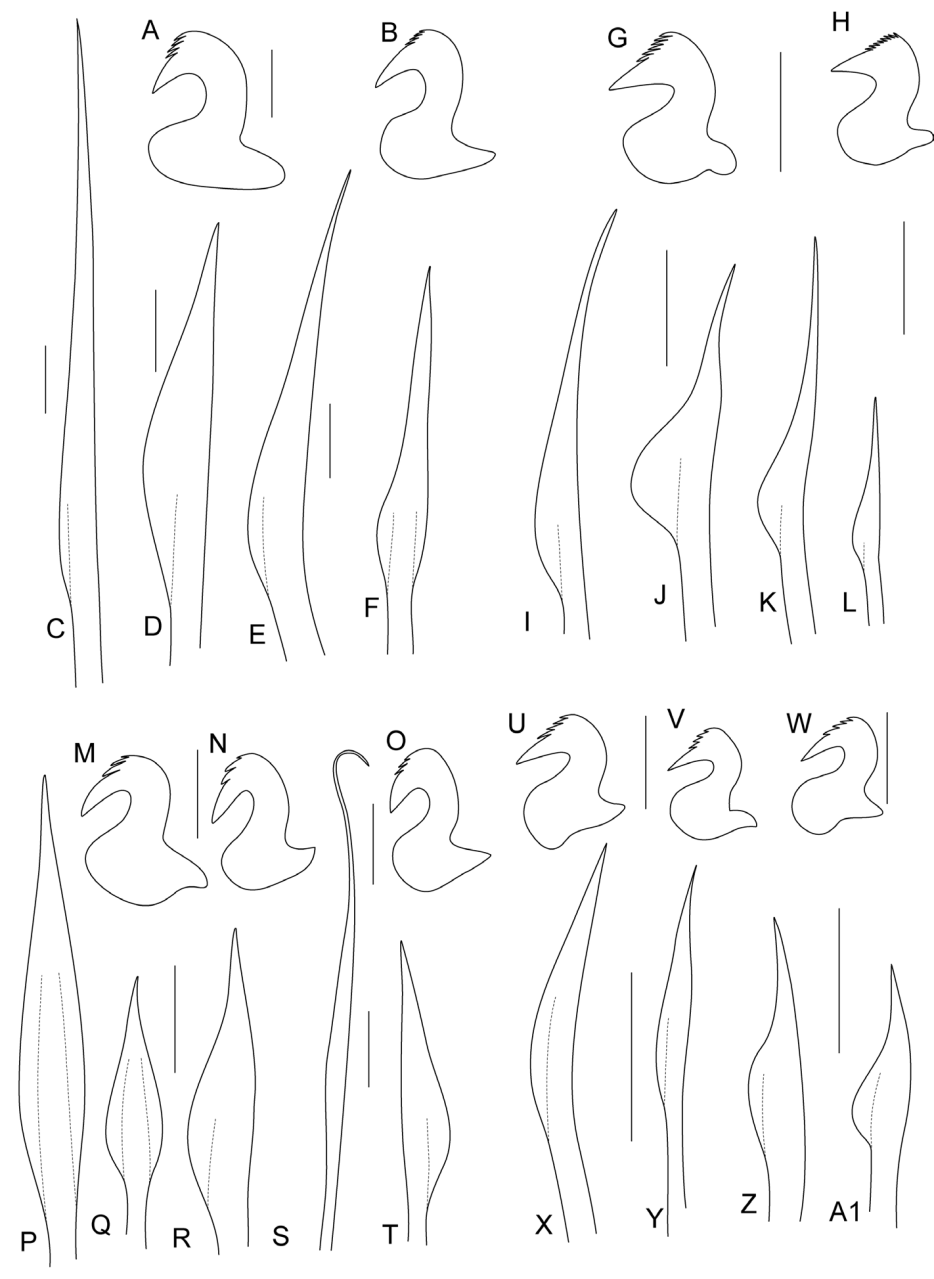
Line drawings of chaetae and uncini of *Pseudobranchiomma* species in Australia; **A**–**F** *Pseudobranchiomma* cf. *Pseudobranchiomma emersoni* **G**–**L** *Pseudobranchiomma* cf. *Pseudobranchiomma orientalis* **M–T** *Pseudobranchiomma pallida* sp. n. **U**–**Z, A1** *Pseudobranchiomma* cf. *Pseudobranchiomma schizogenica*; **A** Thoracic uncinus **B** Abdominal uncinus **C** Superior thoracic chaeta **D** Inferior thoracic chaeta **E, F** Inferior abdominal chaetae **G** Thoracic uncinus **H** Abdominal uncinus **I** Superior thoracic chaeta **J** Inferior thoracic chaeta **K, L** Inferior abdominal chaetae **M, N** Thoracic uncini **O** Abdominal uncinus **P, Q, R** Inferior thoracic chaetae **S** Superior abdominal chaeta **T** Inferior abdominal chaeta **U, V** Thoracic uncini **W** Abdominal uncinus **X, Y** Superior thoracic chaetae **Z, A1** Inferior thoracic chaetae. Scale bars: **A–F** = 2 µm; **G–L** = 4 µm; **M–T** = 2 µm; **U–Z, A1** = 2 µm.

Gravid female, incomplete; body measuring 20 mm long and 2 mm wide, with seven thoracic (Fig. 2D) segments. Crown 8 mm long, slightly involuted ventrally at base, with 17 radioles on each side, connected by an inconspicuous membrane, nearly 1/8^th^ of length of radioles. Radioles with pinnules of constant length along radioles (Fig. 2B, C), shorter distally; tips of radioles as long as pinnules or shorter. Radiolar flanges present, with around 10 short, flattened, flap-like serrations along entire length of radioles (Fig. 2B, C). Radiolar eyes absent. Dorsal lips with tapered radiolar appendages, almost as long as three thoracic segments, with dorsal lamellae attached to base of adjacent radioles. Dorsal pinnular appendage absent. Four rows of vacuolated cells basally supporting radioles. Ventral lips and parallel lamellae present, with prominent ventral sacs directed outside of the radiolar crown (Fig. 2D–F). Collar with wide dorsal gap, margins fused to end of first chaetiger (Fig. 2F); lateral collar margins smooth, just covering junction of crown and thorax (Fig. 2E, F). Ventral lappets large, subtriangular, non-overlapping (Fig. 2D). Ventral shields conspicuous, first shield trapezoidal in shape, but appearing as an anterior Y-shaped and a posterior W-shaped segment when stained with methyl green; shields not in contact with or indented by ventral tori in all thoracic chaetigers (Fig. 2D). Interramal eyespots conspicuous. (Fig. 2D, E). First chaetiger with narrowly hooded chaetae. Rest of thoracic chaetigers with about six superior elongate narrowly hooded chaetae (Fig. 3C) and 16 shorter spine-like inferior chaetae arranged in two rows (Fig. 3D). Neuropodial uncini with approximately five rows of teeth above the main fang, well developed breast and short handle (Fig. 3A). Abdominal chaetigers with narrowly hooded superior chaetae and shorter spine-like chaetae (Fig. 3E) appearing broadly hooded depending on angle (Fig. 3F). Notopodial uncini similar to thoracic ones (Fig. 3B). Pygidium missing. Eggs are present in thorax and anterior abdominal segments.

##### Colour pattern.

Body pale with distinct interramal eyespots of same size in thorax and abdomen (Fig. 2D, E) and purple pigment spots sparsely distributed on thorax (Fig. 2D–F) and dorsally on abdomen. Crown with wide purple band at base (Fig. 2A) and approximately 10 irregular, purple bands (some incomplete transversely) evident on outer side of radioles and flanges; only one yellow band present on distal end of radioles. Dorsal margins of collar (Fig. 2F) and ventral lappets (Fig. 2D) with scattered spots.

##### Remarks.

*Pseudobranchiomma emersoni* Jones, 1962 is a species that was originally described from Jamaica, but has also been reported from the Cape Verde Islands (according to Knight-Jones 1994, p.197, although not verified since) and Florida, USA (Nogueira et al. 2006). It is characterised by a combination of features: up to ten pairs of short flat serrations along each radiole, 5–6 rows of teeth over the main fang in thoracic uncini, a branchial crown that has narrow irregular bands of purple colour (or “splotches”), with often a reduced number of thoracic segments (as few as four, indicating evidence of imperfect regeneration after asexual reproduction), large interramal eyespots, and a collar ventral shield that is trapezoidal in shape (according to Tovar-Hernández and Dean 2014). The Australian specimen, when stained with methyl green, displayed a similar staining pattern on the collar ventral shield as that described for *Pseudobranchiomma schizogenica* Tovar-Hernández and Dean, 2014, although the authors state that this feature differentiates *Pseudobranchiomma schizogenica* from *Pseudobranchiomma emersoni* and *Pseudobranchiomma orientalis* (Tovar-Hernández & Dean, 2014). Although these latter authors also describe the interramal spots of *Pseudobranchiomma emersoni* as “large”, Knight-Jones and Giangrande’s (2003) illustration of the type specimen indicates small spots, so there is some ambiguity regarding this feature, particularly as this can be a subjective assessment. The Australian specimen described above concurs in most respects with *Pseudobranchiomma emersoni*, particularly the flattened step-like form of the radiolar serrations, the irregular colour pattern of the branchial crown and the possession of large interramal eyespots, but because there are slight differences (e.g. 4–5 rows of teeth of the thoracic uncini; the similarity of staining pattern of the ventral shields with *Pseudobranchiomma schizogenica*), and the lack of multiple specimens, we prefer to reserve definite identification until there can be examination of more specimens from Australia, and comparison with the type specimens. The report of this species by Russell and Hewitt (2000) in the ports of Darwin, Northern Territory, is not confirmed, as material from this survey, deposited at the NTM, was examined, and specimens labelled as “*Pseudobranchiomma emersoni*”, were found to be *Pseudobranchiomma* cf. *Pseudobranchiomma schizogenica*.

##### Distribution.

Species known from Jamaica, Florida (USA), Cape Verde Islands, and now Heron Island, Queensland, Australia, where it inhabits coral rubble at shallow depths.

#### Pseudobranchiomma cf. orientalis

Taxon classificationAnimaliaSabellidaSabellidae

(McIntosh, 1885)

**Figure 4.**
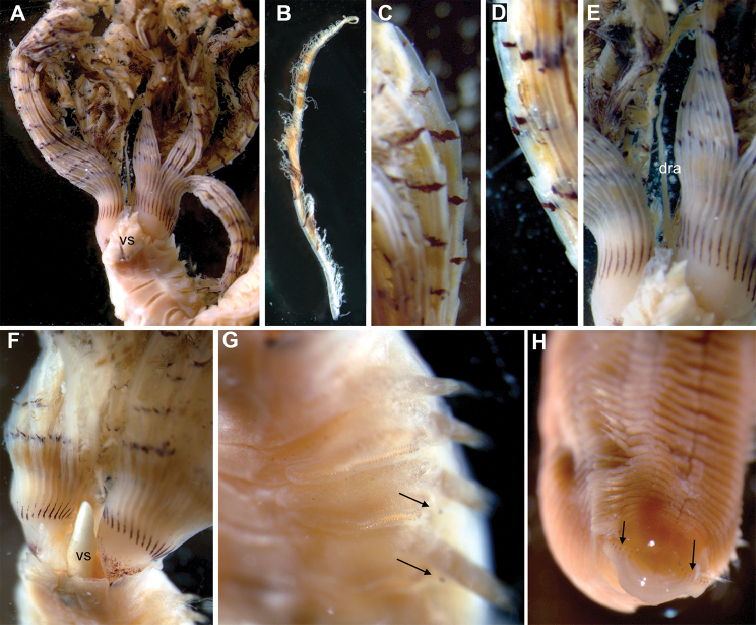
*Pseudobranchiomma* cf. *Pseudobranchiomma orientalis* AM W.32677, AM W.32679: Photographs. **A** Anterior end, ventral view **B** Detached radiole **C** Detail of lateral radiole, mid length **D** Detail of base lateral radiole **E** Detail of radiolar crown base with dorsal radiolar appendages **F** Detail of radiolar crown base with ventral sacs **G** Anterior thoracic parapodia **H** Posterior abdominal segments. dra, dorsal radiolar appendages; vs, ventral sac; black arrows, interramal eyespots.

**Figure 5.**
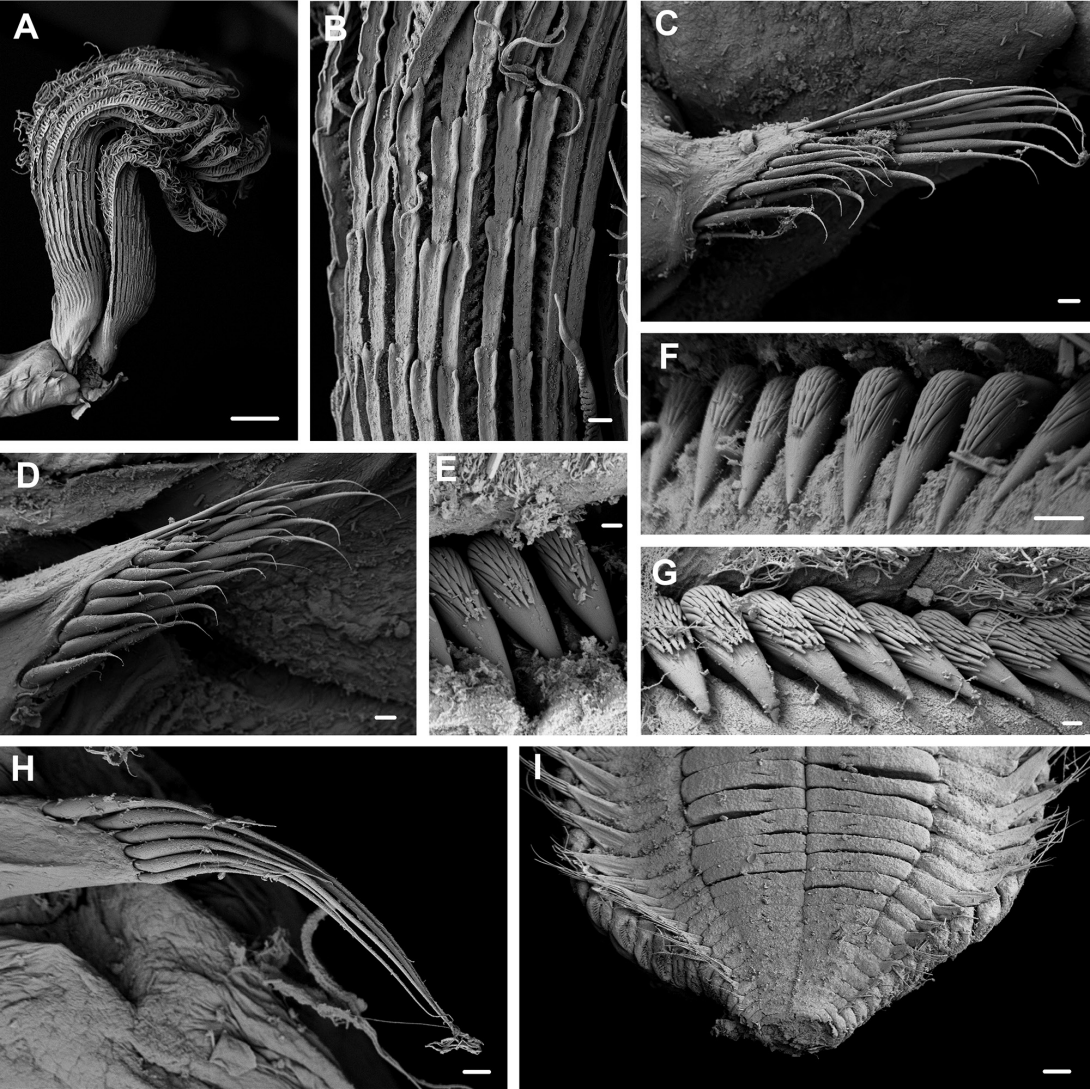
*Pseudobranchiomma* cf. *Pseudobranchiomma orientalis* AM W.37204: Scanning electron micrographs **A** Anterior end, lateroventral view **B** Detail of lateral flanges serrations **C** Notopodia, first thoracic segment **D** Notopodia, fifth thoracic segment **E** Uncini, second thoracic segment **F** Uncini, fifth thoracic segment **G** Posterior abdominal uncini **H** Neuropodia, mid abdominal segment **I** Posterior end, ventral view. Scale bars: **A** = 1 mm; **B, I** = 100 µm; **C, D, G, H** = 20 µm; **E** = 3 µm; **F** = 10 µm.

Figures 3G–L , 4 , 5

? Dasychone orientalis McIntosh, 1885: 498—500, pl. LII, fig 5, Pl.XXXA, figs 19–21, pl. XXXIXA, fig 4.
? Pseudobranchiomma orientalis : Knight-Jones 1994: fig. 4k; Russell and Hewitt 2000: 69, 89; Tovar-Hernández and Dean 2014: 936, table 1.

##### Material examined.

**Australia: Queensland**: AM W.10308 (1 on microscope slide), Calliope River, 23°49'S, 151°13'E, 8 Oct 1975; AM W.37752 (4 specs.), Calliope River, 23°51'S, 151°10'E, 1974; AM W.37204 (1 on 2 SEM pins), same site and date; AM W.32677 (1 spec.), Karumba, 17°29'S, 140°50'E, beam trawl, 1 m, Aug 2000; AM W.37749 (2 specs), Karumba port, Berth 2, 17°29'S, 140°50'E, scraping from pylon, 3m, Aug 2000; AM W.37751 (1 spec.), Karumba, 17°29'S, 140°50'E, benthic sled, 15m, Aug 2000; AM W.32678 (1 spec), Cairns, Wharf 8, 16°53'60"S, 145°48'E, scraping from wharf pile, 7 m, 20 Nov 2001; AM W.32679 (2 specs), Weipa, Lorim Point Wharf, 12°40'S, 141°57'E, scraping from wharf pile, 3 m, Oct 1999; AM W.37750 (1 spec.), Weipa, Lorim Point Wharf, 12°40'S 141°57'E, scraping from wharf pile, 7m, Oct 1999. **Northern Territory**: NTM W017392 (3 specs), Darwin Harbour, Iron Ore Wharf, 12°28'21"S, 130°50'34"E, scrapings from wharf pile, 5–10 m, 1998.

##### Diagnosis.

Ten to 25 serrations evenly distributed along entire length of radiolar flanges, Radiolar eyes absent. Thoracic ventral shields and uncinal tori separated by a small gap. Thoracic and abdominal uncini with 5–7 transverse rows of teeth over main fang. Radiolar crown with broad purple basal band, and approximately 20 transverse purple pigment bands along radioles, interspersed with orange and thin white bands; body with few pigment spots and with small, indistinct interramal eyespots.

##### Description of Australian specimens.

Specimens 5–24 mm long (with 12 mm long crown on longest specimen), 3 mm maximum width; 7–8 thoracic and 50 abdominal chaetigers. Crown strongly involuted ventrally (Figs 4A, 5A), almost forming circle, with 10–30 radioles on each side. Radiolar flanges with conspicuous serrations from end of basal membrane to tip (Figs 4C, D, 5B), 10–25 per radiole (Figs 4A, C, 5A). Radiolar eyes absent. Pinnules decreasing in size distally (Fig. 4B). Radioles supported basally by 6–10 rows of vacuolated cells. Basal membrane as long as one to two thoracic segments, 1/7th–1/8th length of radioles. Dorsal lips with long, thin and pointed radiolar appendage 1/3rd length of crown (Fig. 4A, E). Pinnular appendages absent. Ventral lips nearly half of length of dorsal lips, pointed in shape, attached to 5th or 6th ventral radiole, with large and conical ventral sacs, as long as two thoracic chaetigers, located outside crown (Fig. 4A, F). Collar with wide dorsal gap and dorsal margins reaching end of the first chaetiger; lateral margin of collar smooth, covering junction between crown and thorax with short ventrolateral notch present as oblique incision (Figs 4F, 5A). Ventral lappets large, sub-triangular and rounded tips directed laterally (Fig. 4A, F). First segment as long as remaining thoracic segments. Ventral shields conspicuous, with large intersegmental incisions; thoracic ventral shields similar in width, but some specimens with first two shields wider than subsequent ones. First shield trapezoidal in shape, but appearing as an anterior Y-shape and posterior W-shaped segment when stained with methyl green. Small gap between ventral shields and thoracic tori (Fig. 4A, G). Interramal eyespots small, inconspicuous (Fig. 4G, H). First thoracic notopodia with around 10 superior elongate narrowly hooded chaetae (Fig. 5C) and around 12 short, narrowly hooded chaetae arranged in two rows (Fig. 5C). Subsequent thoracic notopodia with superior elongate narrowly hooded chaetae (Figs 3I, 5D) and shorter spine-like inferior thoracic chaetae arranged in two rows (Figs 3J, 5D). Well-developed thoracic tori with uncini decreasing in size ventrally; uncini with five rows of small teeth over main fang, occupying about half its length, breast well developed, long neck and short handle (Figs 3G, 5E, F). Abdominal chaetigers with superior narrowly hooded chaetae (Fig. 3K) and inferior spine-like chaetae (Figs 3L, 5H). Abdominal uncini with several rows of teeth above main fang, breast well developed and short handle (Figs 3H, 5G). Pygidium bilobed. Tube thick with muddy particles attached. One specimen (AM W.32679) with eggs in mid abdominal chaetigers, others with eggs in thorax.

##### Colour pattern.

Preserved specimens may have few pigment spots on body, with some pigment on end of the faecal groove and dark patches on bases of ventral lappets, internally. Crown with pigments units coinciding with serrations, about 20 thin transverse purple-brown bands on outer side of radioles and flanges, continuing in one or two pinnules, and orange and white bands in between, which may fade (Fig. 4A–F). Longitudinal purple-pigmented midline at bases of each radiole and at ventral and dorsal base of crown may be present (Fig. 4A). Dorsal lips sometimes pigmented; ventral sacs conspicuous due to their white colour (Fig. 4A, F).

##### Remarks.

**Figure 6.**
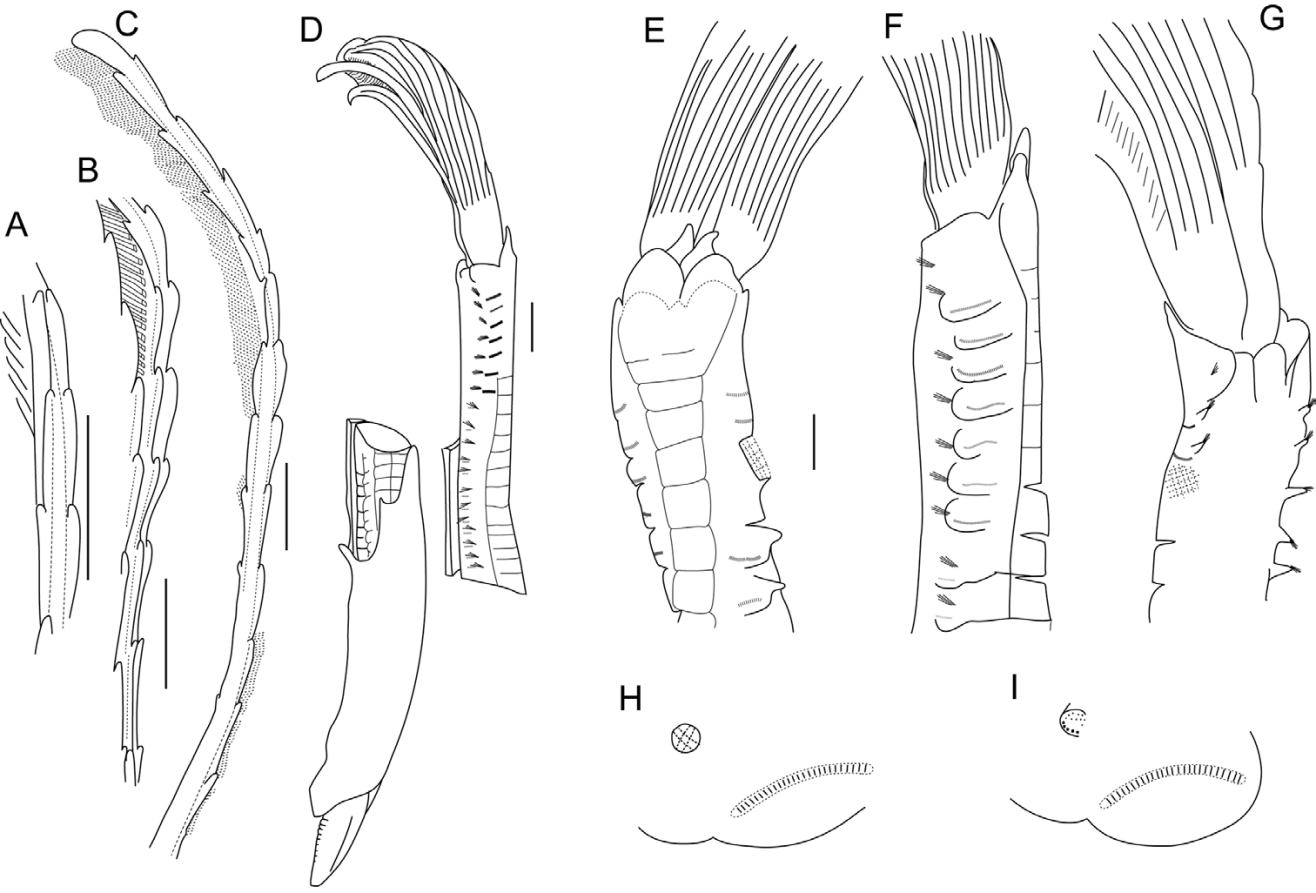
*Pseudobranchiomma orientalis* Type BMNH 85.12.1.393. Line drawings by Phyllis Knight-Jones. **A–C** Second dorsal radiole from different views **D** Holotype, divided in two, and partially covered by the tube, lateroventral view **E** Base of crown and anterior thoracic chaetigers, ventral view **F** Same, lateral view **G** Same, dorsal view **H** Thoracic parapodium **I** Abdominal parapodium. Scale bars: **A–C** = 1 mm; **D** = 2 mm; **E–G** = 1 mm; **H–I** = unknown.

These Australian specimens are identified as *Pseudobranchiomma* cf. *Pseudobranchiomma orientalis*, a species originally described from Hong Kong. Knight-Jones reviewed, and illustrated the types (previously unpublished but shared with MC and reproduced here as Fig. 6) with characters not illustrated in the original description such as the details of the radiolar flanges serrations (Fig. 6A–C), details of the anterior and posterior parts of the body in different views, with special attention to the shape of thoracic ventral shields (fig. 6D–G), and the shape of the parapodia in the thoracic and abdominal segments (Fig. 6H, I) that help confirm the identifications. The Australian specimens share most diagnostic features with the original description and drawings, as well as subsequently published supplementary information about the types (McIntosh 1885, Knight-Jones 1994, Tovar-Hernández and Dean 2014). These are: the number and shape of the serrations in the radiolar flanges (Figs 4C, D, 6A–C), presence of a small gap between the ventral shields and the adjacent thoracic tori (Figs 4G, 6E), and the presence of ventrolateral notches in the collar (Figs 4F, 6E, F) (McIntosh 1885, Knight-Jones 1994). There are some variations however from the reported descriptions, including the presence of interramal eyespots in the thorax, albeit small and inconspicuous, and the staining pattern of the first ventral shield which, as with *Pseudobranchiomma* cf. *Pseudobranchiomma emersoni*, yielded the same result as that reported by Tovar-Hernández and Dean (2014) for *Pseudobranchiomma schizogenica*, even though this was a feature used to differentiate the latter species from *Pseudobranchiomma orientalis* and *Pseudobranchiomma emersoni*. So, because of these differences we prefer to qualify a definite identification of these Australian specimens as *Pseudobranchiomma orientalis*. *Pseudobranchiomma orientalis* was reported in some ports in Northern Territory, Australia (Russell and Hewitt 2000), and after examination of these specimens, it is established that they are *Pseudobranchiomma* cf. *Pseudobranchiomma orientalis*, as described above. Should further sampling and molecular data confirm the status of *Pseudobranchiomma orientalis* in Australia, it is reasonable to suppose that it has been translocated from Asian waters, especially if its restricted range, in ports and harbours, is verified.

##### Distribution.

Pacific Ocean (Hong Kong, Australia: Northern Territory and Queensland).

#### Pseudobranchiomma pallida sp. n.

Taxon classificationAnimaliaSabellidaSabellidae

http://zoobank.org/91A0A21F-530D-48BE-AB44-5DF464BA2E73

Figures 3M–T , 7

##### Type material.

**Australia, Queensland.** Holotype AM W.36366, Heron Island, First Point, North Heron Reef, 23°25'48"S, 151°55'48"E, coral rubble, 13 m, 12 Nov 2009.

##### Diagnosis.

Approximately six pairs of low serrations evenly distributed along radiolar flanges. Radiolar eyes absent. Thoracic ventral shield separated from uncinal tori. Uncini with three transverse rows of teeth over main fang. Radiolar crown with broad purple band at base and distal third with wide yellow band, rest colourless white bands; body pale with distinct interramal eyespots.

##### Description.

**Figure 7.**
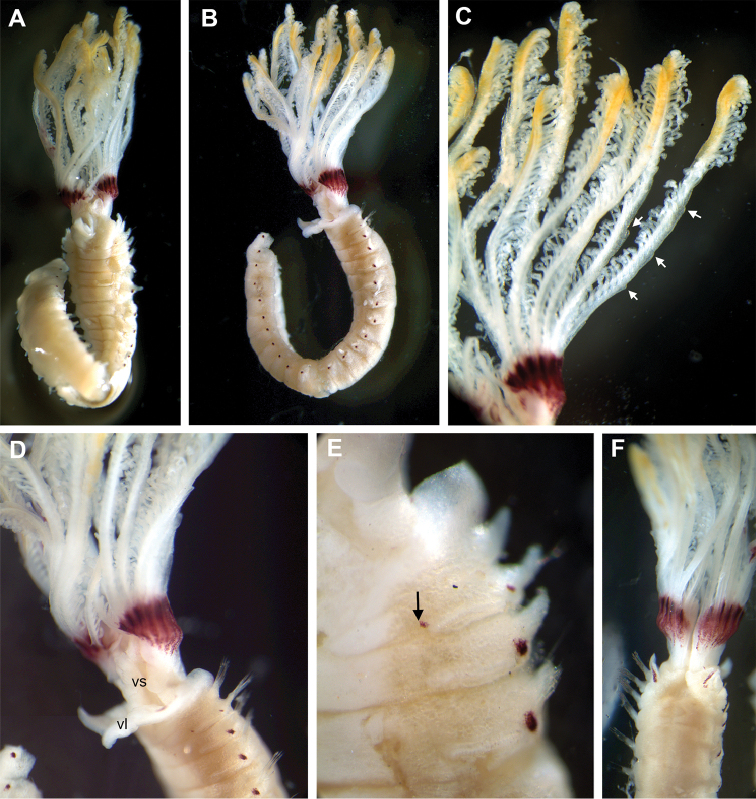
*Pseudobranchiomma pallida* sp. n. AM W.36366: Photographs. **A** Whole specimen, ventral view **B** Whole specimen, lateral view **C** Lateral radioles **D** Detail of base of radiolar crown and anterior segments **E** Anterior thoracic segments, ventrolateral view **F** Anterior end, dorsal view. vl, ventral lappet; vs, ventral sac; white arrows, serrations of radiolar lateral flanges; black arrow, gap between ventral shields and thoracic tori.

Specimen incomplete; body measuring 10 mm long (including crown) and 1 mm wide, with six thoracic and more than 18 abdominal segments (Fig. 7A, B). Crown 4 mm long, slightly involuted at base ventrally, with nine radioles on each side, connected by inconspicuous membrane extending 1/7th–1/8th of radiolar length, or length of one thoracic segment (Fig. 7D). Radiolar flanges present, with about six low serrations along entire length of radioles (Fig. 7C). Radiolar eyes absent (Fig. 7A–C). Radioles supported basally by four rows of vacuolated cells. Radiolar pinnules similar in length, shorter distally; tips of radioles as long as pinnules or shorter (Fig. 7C). Dorsal lips with tapered dorsal radiolar appendages, about as long as two thoracic segments, with dorsal lamella attached to base of adjacent radiole. Dorsal pinnular appendages absent. Ventral lips and parallel lamellae present with prominent ventral sacs directed outside of radiolar crown (Fig. 7A, B, D). Collar margins separated dorsally by wide gap, with dorsal margins fused to end of first chaetiger (Fig. 7F), lateral collar margins smooth, just reaching junction of crown and thorax (Fig. 7E). Ventral lappets, sub-triangular and non-overlapping (Fig. 7D). Ventral shields of first two segments slightly shorter than other thoracic segments (Fig. 7E). First shield trapezoidal in shape, but appearing as anterior Y-shape and posterior W-shaped segment when stained with methyl green. Ventral shields not in contact with or indented by tori (Fig. 7E). Interramal eyespots conspicuous (Fig. 7D, E). First chaetiger with narrowly hooded chaetae arranged in two rows. Rest of thoracic chaetigers with about five superior narrowly hooded chaetae and 8–10 shorter inferior spine-like thoracic chaetae with hood similar width to shaft (Fig. 3R) appearing in some cases as broadly hooded (Fig. 3P, Q). Neuropodial uncini with three rows of teeth above main fang, well-developed breast and short handle (Fig. 3M, N). Abdominal chaetigers with narrowly hooded superior chaetae (Fig. 3S) and spine-like inferior chaetae (Fig. 3T). Notopodial uncini similar to thoracic ones (Fig. 3O). Pygidium missing.

##### Colour pattern.

Body pale with distinct interramal eyespots (Fig. 7B, D, E) and pigment on dorsal margins of collar (Fig. 7F). Crown with broad purple band at base and distal third with wide yellow band, rest colourless (Fig. 7A–C). Ventral sacs and lappets colourless (Fig. 7D).

##### Remarks.

*Pseudobranchiomma pallida* sp. n. is characterised by the remarkable colour pattern of the radiolar crown with a purple basal band and yellow radiolar tips, instead of the characteristic bands, as well as the absence of pigment spots on the body. This species belongs to the artificial Group A of Knight-Jones and Giangrande (2003), members of which possess serrations along the radiolar flanges (Table 3). The number of serrations (4–6) resembles that of *Pseudobranchiomma paraemersoni* and *Pseudobranchiomma schizogenica* (with 3–4 and 6–11 respectively), while other larger species bear nine or more (Table 1 of Tovar-Hernández and Dean 2014). The new species differs from *Pseudobranchiomma paraemersoni* and *Pseudobranchiomma schizogenica* in the morphology of the uncini, with three transverse rows of teeth over the main fang (4–5 and four rows respectively, for *Pseudobranchiomma paraemersoni* and *Pseudobranchiomma schizogenica*; Tovar-Hernández and Dean 2014).

##### Distribution.

Australia (Queensland, Heron Island).

##### Etymology.

This species is named after its colour pigmentation, pale compared with other *Pseudobranchiomma* species, and completely lacking pigment spots on the body.

#### Pseudobranchiomma cf. schizogenica

Taxon classificationAnimaliaSabellidaSabellidae

Tovar-Hernández & Dean, 2014

Figures 3U–Z, A1 , 8 , 9 , 10

Pseudobranchiomma schizogenica Tovar-Hernández & Dean, 2014: 936–945, figs 1–5.

##### Material examined.

**Australia: Queensland**: AM W.36369 (1 spec.), Heron Island, Sykes Reef, 23°25'57"S, 152°02'02"E, coral rubble, 15 m, 13 Nov 2009; AM W.36368 (1 spec.), Heron Island, First Point, 23°25'56"S, 151°56'02"E, coral rubble, 13 m, 12 Nov 2009; AM W.36364 (1 spec.), Sykes Reef, 23°25'57"S, 152°02'02"E, coral rubble, 15 m, 13 Nov 2009; AM W.37753 (2 specs) same locality and date; AM W.32676 (1 spec.) Abbott Point, near Bowen, 19°53'S, 148°05'E, pylon scraping, 8 Jun 1998; W.36978 (1 spec. used for sequencing), Lizard Island, MacGillivray Reef, deep reef slope, 14°39'25"S, 145°28'22"E, coral rubble, 30 m, 4 Sep 2010; AM W.41160 (1 spec.), Reef 14–141 south of South Direction Island, 14°42'31"S, 145°31'53"E, in coarse coral rubble, 15 m, 26 Aug 2010; AM W. AM W.43938 (>25 specs), south east of Lizard Island, reef on north west side of North Direction Island, 14°44'36"S, 145°30'20"E, from sand, 10 m, 15 Aug 2013; AM W. 47698 (1 spec.), Lizard Island group, reef on north eastern side of South Island, 14°42'13"S, 145°27'37"E, coral rubble, 5–12 m, 21 Aug 2013. **Northern Territory**: AM W.37754 (3 specs) Darwin, Lee Point, 12°20.0'S, 130°58.3'E, dead coral washings, 3 m, 11 Jun 1993; ex NTM W017392 (10 specs), Darwin Harbour, Iron Ore Wharf, 12°28'21"S, 130°50'34"E, scrapings from wharf pile, 5–10 m, 1998, originally identified as *Pseudobranchiomma orientalis*; NTM W017392 (part, 2 specs), Darwin Harbour, Iron Ore Wharf, 12°28'21"S, 130°50'34"E, scrapings from wharf pile, 7 m,16 Aug 1998, originally identified as *Pseudobranchiomma* cf. *Pseudobranchiomma emersoni*. **Western Australia**: AM W.37756 (13 specs) Ningaloo Reef, 22°45'19"S, 113°42'40"E, sponge and bryozoa, 15–17 m, 19 May 2009; AM W.37757 (3 specs), Ningaloo Reef, 22°45'19"S, 113°42'40"E, sandstone, 15–17 m, 19 Jun 2009; NTM W018246 (>50 specs) Ashmore Reef, inner lagoon, encrusting sponges, 15 m, 01 Jun 2002, originally identified as *Pseudobranchiomma orientalis*.

##### Comparative material.

**Hawaii**: AM W.35576 (1 spec.), AM W.35577 (1 spec), AM W.35578 (1 spec.), AM W.37206, (1 spec. on SEM pin), AM W.37207 (1 spec. on SEM pin), all from Oahu, Coconut Island, 21°25'48"N, 157°57'43”, epifauna growing on pier, 1 m, 4 Sep 2008.

##### Diagnosis.

Three to six pairs of digitiform radiolar serrations evenly distributed along entire length of radioles. Radiolar eyes absent. Thoracic ventral shields and neuropodial tori separated by a gap. Thoracic and abdominal uncini with about four transverse rows of teeth surmounting main fang. Radiolar crown with transverse dark purple and orange bands at base and 4–6 irregular purple bands along radioles. Body pale, or with some purple patches; large interramal eyespots decreasing posteriorly.

##### Description of Australian specimens.

**Figure 8.**
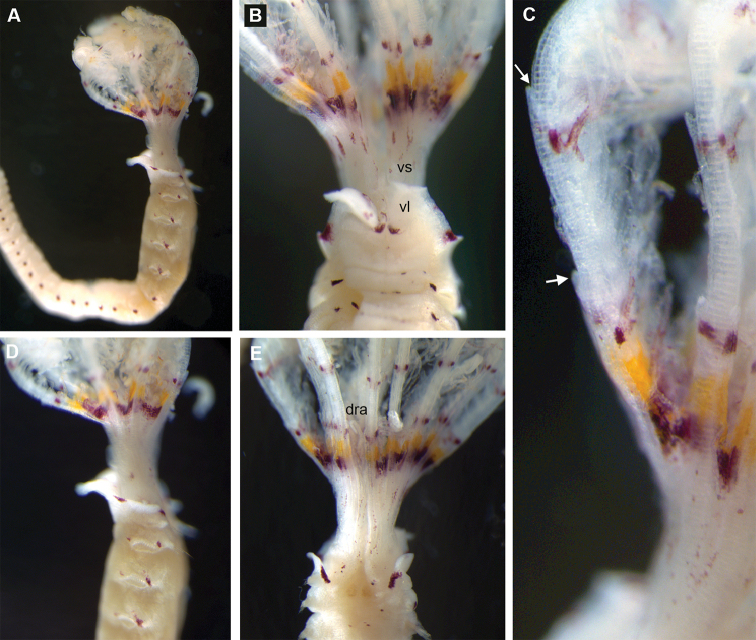
*Pseudobranchiomma* cf. *Pseudobranchiomma schizogenica*. AM W.36368, AM W.36369. Photographs. **A** Anterior end, lateral view **B** Detail of base of crown and anterior segments, ventral view **C** Detail of lateral radiole **D** Anterior thoracic segments, lateral view **E** Anterior thoracic segments, dorsal view **F** Anterior thoracic segments, dorsal view. dra, dorsal radiolar appendages; vl, ventral lappet; vs, ventral sac; white arrows, serrations of radiolar lateral flanges.

**Figure 9.**
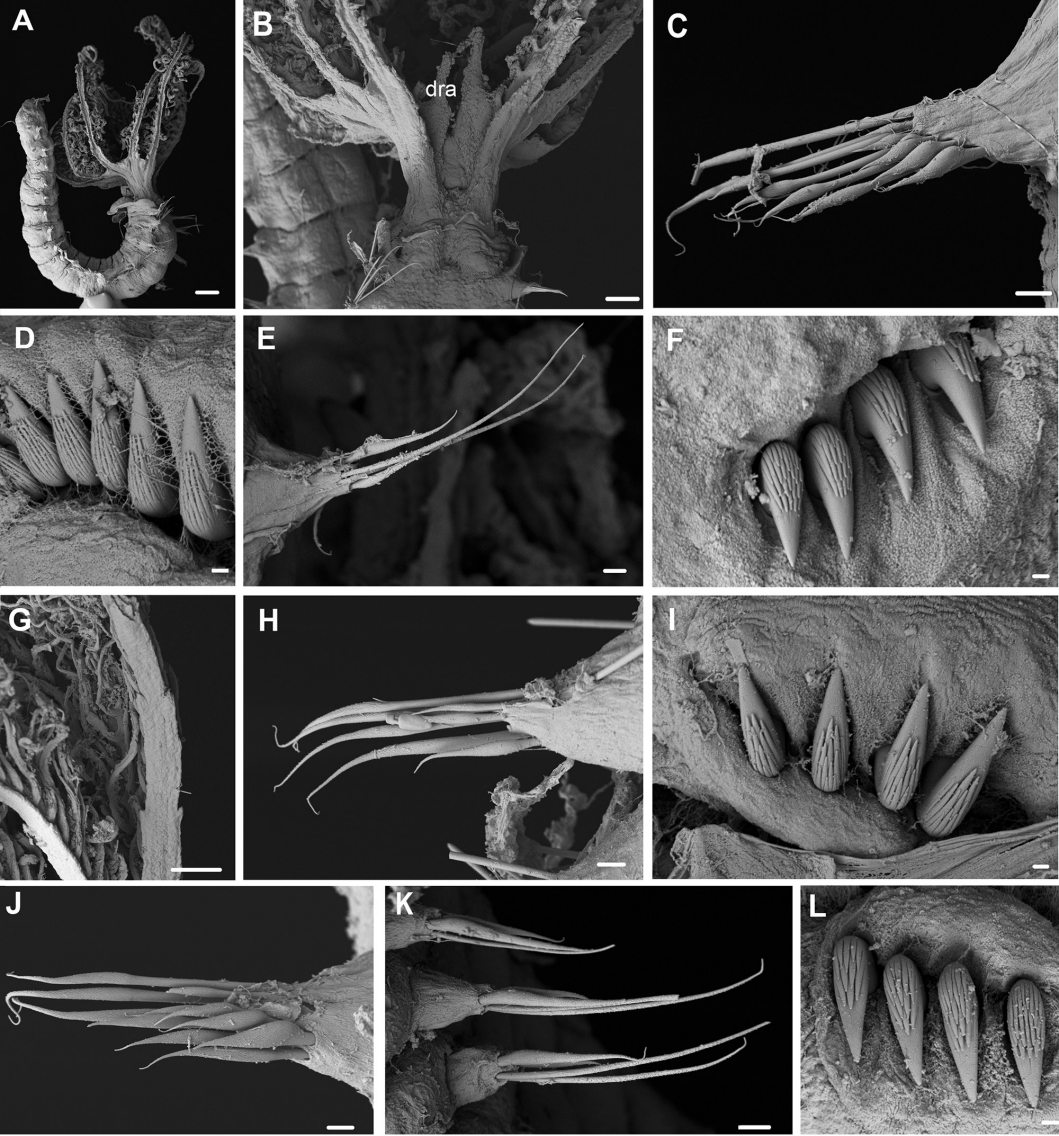
*Pseudobranchiomma* cf. *Pseudobranchiomma schizogenica*. Scanning electron micrographs. **A–F** specimen from Queensland AM W.37205 **G–L** specimen from Western Australia AM W.37203. **A** Whole specimen, lateral view **B** Base of crown, dorsal view with dorsal lips and radiolar appendages **C** Notopodia, second thoracic segment **D** Uncini, third thoracic segment **E** Posterior abdominal neurochaetae **F** Uncini, posterior abdominal segment **G** Detail of lateral radiole with serrations in flanges **H** Notopodia, first thoracic segment **I** Uncini, fifth thoracic segment **J** Notopodia fourth thoracic segment **K** Neuropodia, mid abdominal chaetigers **L** Posterior abdominal uncini. Scale bars: **A** = 200 µm; **B, G** = 100 µm; **C** = 20 µm; **D, F, I, L** = 2 µm; **E, H, J, K** = 10 µm.

**Figure 10.**
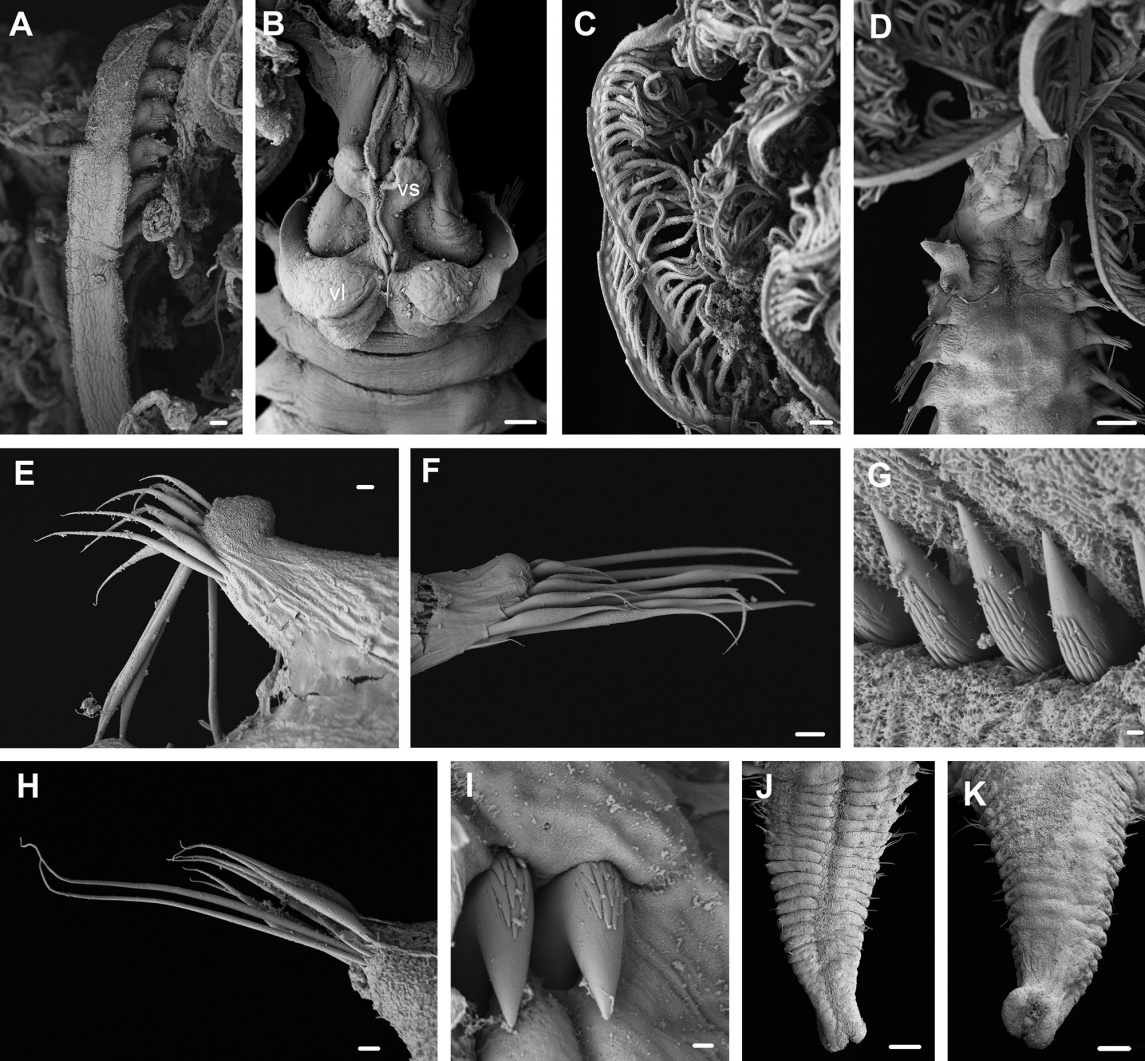
*Pseudobranchiomma* cf. *Pseudobranchiomma schizogenica* from Hawaii AM W.37206, AM W.37207: Scanning electron micrographs. **A** Detail of radiolar flanges serrations **B** Base of crown and anterior segments, ventral view **C** Lateral radioles **D** Anterior segments, dorsal view **E** Notopodia, first thoracic segment **F** Notopodia, second thoracic segment **G** Uncini, third thoracic segment **H** Neuropodia, posterior abdominal segment **I** Posterior abdominal uncini **J** Posterior end, ventral view (pygidium regenerating) **K** Posterior end, dorsal view. vl, ventral lappets; vs, ventral sac. Scale bars: **A** = 20 µm; **B, C, J, K** = 100 µm; **D** = 200 µm; **E, H** = 10 µm; **F** = 20 µm; **G, I** = 2 µm.

Specimens range from 3–19 mm long, 0.2–1 mm wide, with 4–7 thoracic and numerous abdominal segments. One complete specimen from AM W.43938 measures 19 mm in length and 1 mm maximum width, including crown 4 mm long, with 6 thoracic and >80 abdominal chaetigers. Body thin and cylindrical. Crown length varies between 1.5 and 4 mm. Radiolar crown lobes semicircular at base, with about nine radioles on each side, connected by basal membrane equivalent to length of at least one thoracic chaetiger (Figs 8D, 9A, B) or 1/8th of radiolar length. Radioles with serrated radiolar flanges, 3–6 digitiform serrations along entire length of radioles (Figs 8C, 9A, G, 10C). Radiolar eyes absent. Pinnules of constant length along radioles, shorter distally; tips of radioles as long as pinnules or shorter. Radioles supported basally by four rows of vacuolated cells. Dorsal lips with tapered dorsal radiolar appendages, about as long as 3–4 thoracic segments (about one third of radiolar crown length), with dorsal lamella attached to base of adjacent radiole (Fig. 9B). Dorsal pinnular appendages absent (Fig. 9B). Ventral lips and parallel lamellae present, with prominent ventral sacs directed outside of the radiolar crown (Figs 9A, 10B). Collar with dorsal margins separated by wide gap (Figs 8E, 9B, 10D), margins fused to end of first chaetiger; laterally, collar margins smooth, only just reaching junction of crown and thorax (Figs 8E, 9A). Ventral lappets large, sub-triangular, non-overlapping (Figs 8A, B, 9A, 10B). Ventral shields conspicuous (Fig. 8B), first shield trapezoidal, but when stained with methyl green, appears separated into anterior Y-shaped half and posterior W-shaped half; second shield trapezoidal, following shields rectangular. All ventral shields not in contact with or indented by tori. Interramal eyespots conspicuous (Fig. 8A, D). First chaetiger with narrowly hooded chaetae arranged in two rows (Figs 9H, 10E); remaining thoracic chaetigers with about five superior elongate narrowly hooded chaetae and around nine shorter spine-like inferior chaetae (Figs 3X, Y, 9C, J, 10F) with hood as wide as shaft. Neuropodial uncini with about four rows of teeth above main fang (Figs 3U, V, 9D, F, 10G) well-developed breast and short handle with rounded knob on base (Fig. 3U, V). Abdominal chaetigers with narrowly hooded superior chaetae and spine-like inferior chaetae with hood about half width of shaft (Figs 3Z, A1, 9E, K, 10H). Notopodial uncini very similar to thoracic ones (3W, 9F, L, 10I). Pygidium bilobed.

##### Colour pattern.

Body pale with large interramal eyespots (Fig. 8A), decreasing in size gradually towards posterior; small dark purple pigment spots sparsely distributed (Fig. 8A, B, E) over entire body. Some specimens have purple patches on ventral shields as well as further along ventrum and dorsum. Crown with purple pigmentation in basal membrane (Fig. 8A–E), above which there is a pale band followed by dark purple and orange bands (Fig. 8A–E). Pairs of dark purple pigment spots on outer edge of radioles form 4–6 distinct bands along length of radioles (Fig. 8A–E), coinciding with number of serrations along radioles. On some radioles pigmentation extends to base of one or two pinnules. Dorsal margins of collar (Fig. 8E) and ventral lappets (Fig. 8B) have some purple pigmentation. Preserved specimens usually pale with few brown patches on collar and lappets, crowns with some bands of brown pigment, and may have brown longitudinal lines on base of crown. Conspicuous interramal eyespots are maintained after preservation.

##### Remarks.

This species, originally described from the Gulf of California, is characterised by having radioles with short and digitiform serrations along the entire radiolar length, ventral shield of collar trapezoidal and divided into two halves, thoracic superior chaetae and abdominal chaetae with hoods narrower than shafts and thoracic inferior chaetae spine-like with hoods as wide as shafts (Tovar-Hernández and Dean 2014). The specimens found in several localities around the northern Australian coastline and in Hawaii match this diagnosis and additionally share the same colour pattern (four to six repeated pigment units, resembling transverse bands and a wider orange band on the base of radioles) and the number of teeth over the main fang (four). There are, however, some differences between these specimens and the original description of *Pseudobranchiomma schizogenica*, including one feature considered to be diagnostic of this species: the lateral margins of the collar, because they are oblique, do not always cover the anterior peristomial ring (Fig. 8E); there are also fewer flange serrations per radiole (up to six) in the Australian specimens, even the largest ones, compared with the Gulf of California specimens (6–11). The specimens also somewhat resemble *Pseudobranchiomma paraemersoni* Nogueira, Rossi and Lopez, 2006, from Brazil, a species that also shares a similar number of thoracic segments (between 5 and 7), large interramal eyespots, and the typical transverse bands of pigments in radiolar crown, but differs from *Pseudobranchiomma* cf. *Pseudobranchiomma schizogenica* by having fewer number of flange serrations per radiole (3–4) and inferior thoracic spine-like chaetae with hood twice as wide as shaft (hood only as wide as shaft in *Pseudobranchiomma schizogenica*). Similarly, *Pseudobranchiomma emersoni* shares some features with *Pseudobranchiomma* cf. *Pseudobranchiomma schizogenica*, but differs from it by the irregular branchial crown pigment pattern, the greater number of rows of teeth above main fang in thoracic uncini (5–6), greater number of radioles (14), and the non-differentiated first ventral shield.

##### Distribution.

Southern Gulf of California (Mexico), northern Australia and Hawaii. This species is associated with coral rubble and epifauna attached to hard substrates in shallow depths (0–15 m).

### Key to species of *Pseudobranchiomma*

The number of *Pseudobranchiomma* species considered as currently valid (17) follows Knight-Jones and Giangrande (2003) but includes subsequently described species. This key is based largely on descriptions in the literature, and most of them do not include intraspecific variation, so caution should be taken if specimens diverge from statements in the key. Old descriptions also lack enough relevant information to clearly separate species. Therefore, such points of weakness in the key are marked with an asterisk (*).

**Table:** 

1	Radioles with distinct, paired, serrated flanges	**2**
–	Radioles with flanges reduced to low ridges (lacking distinct serrations)	**9**
2	Serrations distinct along most (or all) length of radioles	**3**
–	Serrations only distinct on distal parts of radioles	**7**
3	Radioles with paired compound eyes present	***Pseudobranchiomma grandis* (Baird, 1865)** (New Zealand) (Fig. 11) or *reportedly present ***Pseudobranchiomma serratibranchis* (Grube, 1878)** (Philippines)
–	Radioles without distinct radiolar eyes	**4**
4	Radioles with over 10 pairs of serrations on lateral flanges	**5**
–	Radioles with maximum of 10 pairs of serration on lateral flanges	**6**
5	Radioles with up to 25 serrations and coloured transverse bands; thorax generally with 8 thoracic chaetigers; thoracic uncini with 6–7 rows of teeth	***Pseudobranchiomma orientalis* (McIntosh, 1885)** (Hong Kong)
–	Radioles with 13–19 serrations and 10–19 transverse pigmented bands; thorax with 6–10 thoracic chaetigers; 4–5 rows of teeth in thoracic uncini	***Pseudobranchiomma paulista*Nogueira et al., 2006** (Brazil)
6	Radiolar crown without pigmented transverse dark bands; radiolar lobes pigmented with purple and radioles white with yellow tips. Radioles with six serrations along their length; three rows of teeth above main fang of thoracic uncini	***Pseudobranchiomma pallida* sp. n.** (Australia)
–	Radiolar crown with several pigmented transverse bands (regular or irregular)	**7**
7	Radioles with up to 10 serrations and 10 narrow irregular purple bands; thorax with 4–8 chaetigers; 5–6 rows of teeth above main fang of thoracic uncini	***Pseudobranchiomma emersoni* Jones, 1962** (Caribbean)
–	Radioles with 3–4 serrations and transverse bands (purple and yellow; a few white); thorax with 4–5 thoracic chaetigers; 4–5 rows of teeth above main fang of thoracic uncini	***Pseudobranchiomma paraemersoni*Nogueira et al., 2006** (Brazil)
–	Radioles with 6–11 serrations and 4–6 transverse bands (of purple-orange-white); four rows of teeth above main fang of thoracic uncini; lateral margins of collar oblique and covering anterior peristomial ring	***Pseudobranchiomma schizogenica*Tovar-Hernández and Dean 2014** (Gulf of California)
8	Radiolar eyes reportedly* present	***Pseudobranchiomma odhneri* (Fauvel, 1921)** (Madagascar) or* ***Pseudobranchiomma bocki* (Johansson, 1922)** (Japan)
–	Radiolar eyes absent	**8**
9	Radiolar crown with 12 dark pigment bands (and 7 wide yellow bands between)	***Pseudobranchiomma tricolor* (Grube, 1881)** (Japan)
–	Radiolar crown whitish, darker at base, lacking transverse pigmented bands; thorax with eight thoracic chaetigers; thoracic uncini with over five rows of teeth	***Pseudobranchiomma zebuensis* (McIntosh, 1885)** (Philippines)
10	Peristomial collar fused dorsally to sides of faecal groove	***Pseudobranchiomma punctata* (Treadwell, 1905)** (Hawaii)
–	Collar with free dorsal margins, widely separated from faecal groove	**10**
11	Radioles with paired compound eyes	**11**
–	Radioles without distinct compound eyes (may have granular pigment patches)	**12**
12	Thorax broader than long (with up to 8 thoracic chaetigers); each side of crown in spiral of up to 5 whorls (mature specimens)	***Pseudobranchiomma longa* (Kinberg, 1867)** (South Africa)
–	Thorax longer than broad (with up to 13 thoracic chaetigers); radiolar lobes never spiralled	***Pseudobranchiomma perkinsi* Knight-Jones & Giangrande, 2003** (Florida)
13	Thorax with 4–6 segments; first thoracic chaetiger less than 1.5 times length of the following ones	***Pseudobranchiomma minima* Nogueira & Knight-Jones, 2002** (Brazil)
–	Thorax with 8 segments; first thoracic chaetiger 2–3 times length of the following ones	***Pseudobranchiomma tarantoensis* Knight-Jones & Giangrande, 2003** (Italy)

**Figure 11.**
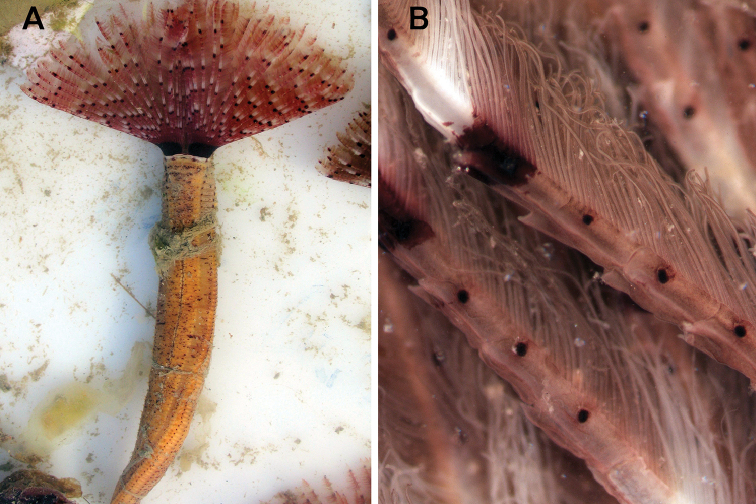
*Pseudobranchiomma grandis* from New Zealand. **A** Whole live animal **B** Section of radioles, showing paired radiolar eyes and serrations of lateral flanges. Photos by Rod Asher.

## Discussion and conclusions

The genus *Pseudobranchiomma* was erected based on the short thorax (with less than the usual eight thoracic chaetigers), absence of compound radiolar eyes (unlike members of *Branchiomma* and some *Bispira*), and the presence of ‘reduced stylodes’ (Jones 1962), that are now considered to be serrations of the radiolar flanges (Knight-Jones and Giangrande 2003), features that have been recognised not to be unique to members of the genus and also not shared by all congeners (e.g. Fitzhugh 1989, Knight-Jones 1994, Fitzhugh and Rouse 1999, Nogueira and Knight-Jones 2002, Knight-Jones and Giangrande 2003, Nogueira et al. 2006, Capa 2008). It is therefore not surprising that monophyly of the genus has not been confirmed after our analyses of morphological data. The low number of sequences available for this study does not allow to assess its’ monophyly either. However, and contrary to results obtained after analyses of morphological data, DNA sequences suggest that *Branchiomma* is not nested within *Pseudobranchiomma* or sister to it (Fitzhugh 1989, Capa 2008, Nogueira et al. 2010), and instead, *Sabellastarte* and *Sabella* are the closest related taxa. It is in fact difficult to discern between members of *Sabellastarte* (especially if small) and those *Pseudobranchiomma* without radiolar flanges and eyes. The only attribute to distinguish between members of these two genera in these cases, is the position of the ventral sacs, which are inside the radiolar crown in members of *Sabellastarte* and outside the crown in members of *Pseudobranchiomma* (e.g. Knight-Jones and Mackie 2003, Capa et al. 2010).

Relationships within the genus indicate that the groups proposed by Knight-Jones and Giangrande (2003) based on characteristics of the radiolar crown (presence of eyes, flanges and serrations), although still valuable for comparing similar-looking species do not seem to hold any phylogenetic information.

In this study, a new species, *Pseudobranchiomma pallida* sp. n., is herein described, and another species, *Pseudobranchiomma* cf. *Pseudobranchiomma schizogenica* is reported in Australia for the first time, an indication that the group is more diverse than previously considered. Nevertheless, this diversity could be due, in part, to unintentional translocations. Some specimens in this study could be assigned to three species, *Pseudobranchiomma* cf. *Pseudobranchiomma emersoni*, *Pseudobranchiomma* cf. *Pseudobranchiomma orientalis* and *Pseudobranchiomma* cf. *Pseudobranchiomma schizogenica*, originally described from distant and disjunct geographic areas (Jamaica, Southern Gulf of California and Hong Kong, respectively) but also reported from other worldwide localities (Knight-Jones 1994, Russell and Hewitt 2000, Swami and Udhayakumar 2010, Tovar-Hernández and Dean 2014). The hypothesis of these species being translocated requires testing. Due to the great morphological similarity displayed by individuals from such disjunct populations, the use of molecular markers could be an effective method to test whether they belong to the same species. Another issue to be resolved, if the translocations are indeed corroborated, would be the actual origin of the species. At this juncture, it is uncertain whether they have been introduced to Australia or were translocated from this continent. The fact that *Pseudobranchiomma* cf. *Pseudobranchiomma orientalis* is mainly reported from port environments in Australia may possibly be an indication of its introduction herein. *Pseudobranchiomma schizogenica* was originally collected in 2011 from marinas in Mexico as part of fouling communities (Tovar-Hernández and Dean 2014) and therefore it seems possible that it is also not its natural distribution range but the species has also been introduced there. In Australia, *Pseudobranchiomma* cf. *Pseudobranchiomma schizogenica* was collected from Darwin ports as early as 1998, possibly introduced, and may have spread to more pristine, non-port areas in Queensland and Western Australia. It has been found in Hawaii also, although not in a major port environment, but in the environs of a yachting port-of-call. Further analysis using molecular data of more specimens collected from around the world may eventually lead to some clarification of its true origin. Colonies of *Pseudobranchiomma schizogenica* (or *Pseudobranchiomma* cf. *Pseudobranchiomma schizogenica*) now appear to be abundant in fouling communities of marinas and ports, and may possibly be a potential pest species.

## Supplementary Material

XML Treatment for Pseudobranchiomma

XML Treatment for Pseudobranchiomma cf. emersoni

XML Treatment for Pseudobranchiomma cf. orientalis

XML Treatment for Pseudobranchiomma pallida

XML Treatment for Pseudobranchiomma cf. schizogenica
