# Corrigenda: Capa M, Murray A (2017) Combined morphological and molecular data unveils relationships of *Pseudobranchiomma* (Sabellidae, Annelida) and reveals higher diversity of this intriguing group of fan worms in Australia, including potentially introduced species. ZooKeys 622: 1–36. https://doi.org/10.3897/zookeys.622.9420

**DOI:** 10.3897/zookeys.722.21609

**Published:** 2017-12-14

**Authors:** María Capa, Anna Murray

**Affiliations:** 1 NTNU University Museum, Norwegian University of Science and Technology, NO-7491 Trondheim, Norway; 2 Australian Museum Research Institute, 1 William St, Sydney, 2010, NSW, Australia

Several errors related to the numbering in the dichotomous key appearing in the above paper came to our attention after our manuscript was published. The correct numbering to enable accurate identification of the world *Pseudobranchiomma* species is provided here.

## Key to species of *Pseudobranchiomma*

The number of *Pseudobranchiomma* species considered as currently valid (17) follows Knight-Jones and Giangrande (2003) but includes subsequently described species. This key is based largely on descriptions in the literature, and most of them do not include intraspecific variation, so caution should be taken if specimens diverge from statements in the key. Old descriptions also lack enough relevant information to clearly separate species. Therefore, such points of weakness in the key are marked with an asterisk (*).

**Table:** 

1	Radioles with distinct, paired, serrated flanges	**2**
–	Radioles with flanges reduced to low ridges (lacking distinct serrations)	**10**
2	Serrations distinct along most (or all) length of radioles	**3**
–	Serrations only distinct on distal parts of radioles	**8**
3	Radioles with paired compound eyes present	***P. grandis* (Baird, 1865)** (New Zealand) (Fig. 11) or *reportedly present ***P. serratibranchis* (Grube, 1878)** (Philippines)
–	Radioles without distinct radiolar eyes	**4**
4	Radioles with over 10 pairs of serrations on lateral flanges	**5**
–	Radioles with maximum of 10 pairs of serration on lateral flanges	**6**
5	Radioles with up to 25 serrations and coloured transverse bands; thorax generally with eight thoracic chaetigers; thoracic uncini with 6–7 rows of teeth	***P. orientalis* (McIntosh, 1885)** (Hong Kong)
–	Radioles with 13–19 serrations and 10–19 transverse pigmented bands; thorax with 6–10 thoracic chaetigers; 4–5 rows of teeth in thoracic uncini	***P. paulista* Nogueira et al., 2006** (Brazil)
6	Radiolar crown without pigmented transverse dark bands; radiolar lobes pigmented with purple and radioles white with yellow tips. Radioles with six serrations along their length; three rows of teeth above main fang of thoracic uncini	***P. pallida* sp. n.** (Australia)
–	Radiolar crown with several pigmented transverse bands (regular or irregular)	**7**
7	Radioles with up to 10 serrations and 10 narrow irregular purple bands; thorax with 4–8 chaetigers; 5–6 rows of teeth above main fang of thoracic uncini	***P. emersoni* Jones, 1962** (Caribbean)
–	Radioles with 3–4 serrations and transverse bands (purple and yellow; a few white); thorax with 4–5 thoracic chaetigers; 4–5 rows of teeth above main fang of thoracic uncini	***P. paraemersoni* Nogueira et al., 2006** (Brazil)
–	Radioles with 6–11 serrations and 4–6 transverse bands (of purple-orange-white); four rows of teeth above main fang of thoracic uncini; lateral margins of collar oblique and covering anterior peristomial ring	***P. schizogenica* Tovar-Hernández & Dean, 2014** (Gulf of California)
8	Radiolar eyes reportedly* present	***P. odhneri* (Fauvel, 1921)** (Madagascar) or* ***P. bocki* (Johansson, 1922)** (Japan)
–	Radiolar eyes absent	**9**
9	Radiolar crown with 12 dark pigment bands (and 7 wide yellow bands between)	***P. tricolor* (Grube, 1881)** (Japan)
–	Radiolar crown whitish, darker at base, lacking transverse pigmented bands; thorax with eight thoracic chaetigers; thoracic uncini with over five rows of teeth	***P. zebuensis* (McIntosh, 1885)** (Philippines)
10	Peristomial collar fused dorsally to sides of faecal groove	***P. punctata* (Treadwell, 1905)** (Hawaii)
–	Collar with free dorsal margins, widely separated from faecal groove	**11**
11	Radioles with paired compound eyes	**12**
–	Radioles without distinct compound eyes (may have granular pigment patches)	**13**
12	Thorax broader than long (with up to eight thoracic chaetigers); each side of crown in spiral of up to five whorls (mature specimens)	***P. longa* (Kinberg, 1867)** (South Africa)
–	Thorax longer than broad (with up to 13 thoracic chaetigers); radiolar lobes never spiralled	***P. perkinsi* Knight-Jones & Giangrande, 2003** (Florida)
13	Thorax with 4–6 segments; first thoracic chaetiger less than 1.5 times length of the following ones	***P. minima* Nogueira & Knight-Jones, 2002** (Brazil)
–	Thorax with eight segments; first thoracic chaetiger 2–3 times length of the following ones	***P. tarantoensis* Knight-Jones & Giangrande, 2003** (Italy)

